# Enhancing Industrial Hemp (*Cannabis sativa*) Leaf By-Products: Bioactive Compounds, Anti-Inflammatory Properties, and Potential Health Applications

**DOI:** 10.3390/ijms26020548

**Published:** 2025-01-10

**Authors:** Luisa Frusciante, Michela Geminiani, Behnaz Shabab, Tommaso Olmastroni, Neri Roncucci, Pierfrancesco Mastroeni, Laura Salvini, Stefania Lamponi, Alfonso Trezza, Annalisa Santucci

**Affiliations:** 1 Department of Biotechnology, Chemistry and Pharmacy, University of Siena, Via Aldo Moro, 53100 Siena, Italy; luisa.frusciante@unisi.it (L.F.); b.shabab@student.unisi.it (B.S.); tommaso.olmastroni@student.unisi.it (T.O.); p.mastroeni@student.unisi.it (P.M.); stefania.lamponi@unisi.it (S.L.); alfonso.trezza2@unisi.it (A.T.); annalisa.santucci@unisi.it (A.S.); 2 SienabioACTIVE, University of Siena, Via Aldo Moro, 53100 Siena, Italy; 3 Tenuta di Mensanello, Località Mensanello, 34, 53034 Colle di Val d’Elsa, Italy; neri.roncucci@gmail.com; 4 Fondazione Toscana Life Sciences, Strada del Petriccio e Belriguardo, 53100 Siena, Italy; l.salvini@toscanalifesciences.org; 5 ARTES 4.0, Viale Rinaldo Piaggio, 34, 56025 Pontedera, Italy

**Keywords:** *Cannabis sativa*, waste repurposing, cannabinoids, flavonoids, UPLC-MS/MS, inflammation, RAW 264.7, MAPK, osteoarthritis, docking and molecular dynamics simulation

## Abstract

The sustainable utilization of biomass-derived bioactives addresses the growing demand for natural health products and supports sustainable development goals by reducing reliance on synthetic chemicals in healthcare. *Cannabis sativa* biomass, in particular, has emerged as a valuable resource within this context. This study focuses on the hydroethanolic extract of *C. sativa* leaves (CSE), which exhibited significant levels of phenolic compounds contributing to robust antioxidant activity. Evaluation using potassium ferricyanide, ABTS, and DPPH methods revealed potent radical scavenging activity comparable to the Trolox standard. UPLC-MS/MS profiling identified cannabinoids as the predominant secondary metabolites in CSE, with flavonoids also present in substantial quantities. This study investigated the anti-inflammatory potential of CSE on RAW 264.7 macrophages and IL-1β-stimulated C-20/A4 immortalized human chondrocytes, demonstrating protective effects without cytotoxic or mutagenic effects. Mechanistically, CSE reduced inflammation by inhibiting the MAPK and NF-κB signaling pathways. In silico approaches showed the ability of CSE’s main metabolites to bind and influence MAPK and NF-κB activity, confirming in vitro evidence. Incorporating *C. sativa* leaf extract into a hyaluronic acid-based formulation showed biotechnological promise for treating joint inflammation. Future research should aim to elucidate the molecular mechanisms underlying these effects and explore the potential of CSE-derived compounds in mitigating osteoarthritis progression. This approach highlights the significance of utilizing annually increasing biomass waste for sustainable bioactivity and environmental impact reduction.

## 1. Introduction

In the context of the circular bioeconomy, the recovery and utilization of waste biomass from agricultural and marine sources presents a novel opportunity to enhance sustainability and resource efficiency. By transforming overlooked resources such as agricultural residues, we can convert waste biomass into biofuels, biochemicals, bioplastics, and other high-value materials. This shift allows us to transition from an outdated “linear” system to a closed-loop approach where waste is repurposed into useful products. Furthermore, products derived from plant biomass are increasingly recognized for their potential to advance global health sectors [1,2]. Bioactive compounds extracted from plant biomass, including polyphenols, flavonoids, and essential oils, exhibit powerful antioxidant, anti-inflammatory, and antimicrobial properties [3,4,5,6]. Extensively studied for their therapeutic benefits, these bioactives can be utilized in pharmaceuticals, nutraceuticals, and functional foods to address diseases associated with oxidative stress and improve overall health [7,8,9,10,11,12,13,14,15]. This strategic approach holds promise in addressing contemporary issues related to inflammatory conditions while simultaneously addressing environmental challenges.

The sustainable production of biomass-derived bioactives meets the rising consumer demand for natural health products and aligns with sustainable development objectives, fostering ecological balance and reducing dependence on synthetic chemicals in healthcare [16]. Within this framework, industrial *Cannabis sativa* biomass has emerged as a valuable resource, supporting the sustainable progress of second-generation biorefineries. These facilities emphasize the utilization of non-food biomass sources to enhance sustainability practices [17]. *C. sativa* L., commonly known as “weed” or “hemp”, is a versatile plant belonging to the *Cannabaceae* family, which also includes the genus *Humulus*. Known for its extensive history of human use spanning thousands of years, it has been cultivated for its fibers, seeds, and medicinal properties [18]. The taxonomy of *C. sativa* has evolved over time. Initially described as a single species in 1753 by Carl Linnaeus, it was later divided into two species by Jean-Baptiste Lamarck in 1785: *C. sativa*, a taller, fibrous plant, and *C. indica*, a shorter, psychoactive variety [19]. *C. ruderalis* was subsequently proposed by Janischevsky [20]. However, further studies by Small and Cronquist in 1976 consolidated all *Cannabis* plants into a single species, *C. sativa*, encompassing various subspecies and varieties [21].

*C. sativa* populations are categorized based on the ratio of their two main cannabinoids, Δ^9^-tetrahydrocannabinol (Δ^9^-THC or THC) and cannabidiol (CBD). Chemotype I includes THC-dominant plants associated with recreational and medicinal use, while chemotype III refers to CBD-dominant varieties, often called industrial hemp. Chemotype II represents populations with nearly equal THC and CBD levels [22]. Minor chemotypes include chemotype IV, rich in cannabigerol (CBG), and chemotype V, which lacks cannabinoids [23].

Industrial hemp, used predominantly for agricultural and industrial purposes, is distinguished by its low levels of THC, typically less than 1%, making it unsuitable for recreational use [17]. Recent attention has increasingly focused on the industrial applications of *C. sativa* in biofuels, bioplastics, and pharmaceuticals, underscoring its potential contributions to sustainable agriculture. Cultivating *C. sativa* not only addresses concerns about non-renewable resource use [24], but also offers a unique opportunity through the utilization of waste by-products generated during its industrial processing.

While it has been utilized for centuries for medicinal purposes, all *C. sativa* by-products, including hurds, leaves, and inflorescences, which constitute the majority of the plant’s biomass, can be regarded as high-value products [25,26,27]. The primary pharmacologically active compounds in *C. sativa* are the psychoactive THC and nonpsychoactive cannabidiol (CBD), which have been extensively researched due to their potent antioxidant and anti-inflammatory properties [28,29,30,31,32,33,34,35]. Specifically, intriguing therapeutic properties have been identified for managing inflammatory conditions. In recent years, there has been a notable increase in inflammatory pathologies, posing significant challenges to public health worldwide. Conditions such as rheumatoid arthritis, inflammatory bowel diseases (such as Crohn’s disease and ulcerative colitis), psoriasis, and osteoarthritis (OA) are among the most problematic. OA, in particular, is a prevalent chronic condition characterized by the degeneration of joint cartilage and underlying bone, leading to pain, stiffness, and reduced mobility [36]. This rise in inflammatory diseases has sparked intensive research into novel therapeutic approaches, including the exploration of cannabinoids like THC and CBD due to their recognized anti-inflammatory properties [28,30,34,35,37]. These compounds have shown promise in preclinical and clinical studies for mitigating inflammation and alleviating associated symptoms in various inflammatory conditions, offering potential new avenues for treatment and management. Previous studies have suggested that cannabinoids have the potential to directly alleviate pain in synovial joints [38,39,40,41]. Evidence suggests that cannabinoids could act on the endocannabinoid system within arthritic joints and relieve inflammatory and arthritic joint disease [42]. Furthermore, preclinical studies have indicated that cannabinoids can reduce inflammation and pain in animal models with arthritis [43,44,45].

Alongside the dominant phytocannabinoids found primarily in the plant’s inflorescences and seeds, various specialized metabolites like flavonoids and steroids play a significant role in the diverse medicinal properties of *C. sativa* [46]. These bioactives, notable for their antioxidant and anti-inflammatory properties, can also be derived from *C. sativa* waste produced after industrial processing. However, despite their potential, few studies have investigated using *C. sativa* by-products, such as leaves, as a source of phytocannabinoids and other bioactive compounds.

This study aimed to investigate the potential antioxidant and anti-inflammatory effects of a bioactives-enriched extract derived from *C. sativa* leaves collected in Tuscany, Italy. We hypothesized that *C. sativa* by-products could serve as an alternative source of bioactive compounds that can be beneficial in treating patients with inflammatory conditions, including OA.

## 2. Results

### 2.1. Chemical Composition and Antioxidant Capacity of C. sativa Leaves Extract

The *C. sativa* extract (CSE) under investigation was produced using heat-reflux extraction with a 70:30 *v*/*v* ethanol–water mixture, yielding 1.31 ± 0.56 g (or 13.1% *w*/*w*) of dry extract. CSE was characterized for its total phenolic content (TPC) and total flavonoid content (TFC) through spectrophotometric assays (Table 1). The phenolic compounds found in CSE contributed to a reducing power (RP), measured by the potassium ferricyanide method, of 58.50 ± 2.4 mg AAE/g of dry extract. Additionally, the radical scavenging ability was assessed using 2,2′-Azino-bis(3-ethylbenzothiazoline-6-sulfonic acid (ABTS) and 2,2-Diphenyl-1-picrylhydrazyl (DPPH) radicals. In both cases, the extract showed excellent scavenging capacity, with an IC50 for ABTS only slightly higher than that of Trolox (119.13 ± 3.12 µg/mL, *p* = 0.0226), a water-soluble vitamin E analog used as a reference standard.

CSE was further analyzed using UPLC-MS/MS, leading to the identification of 88 metabolites through Compound Discoverer 3.3 software integrated with the ChemSpider database and mzCloud. These findings were validated by comparing the detected metabolites with data from prior metabolomic studies on *C. sativa* metabolites [25,26,47,48]. Table 2 reports the most representative metabolites (>0.1% peak area%) found in CSE, along with their retention time, molecular formulas, calculated MW, *m*/*z*, and error (ppm). The full list of identified compounds is provided in Table S1. For all matched compounds, the error was less than 5 ppm.

### 2.2. CSE Reduced LPS-Induced Inflammation in RAW 264.7 Cells

**Figure 1:**
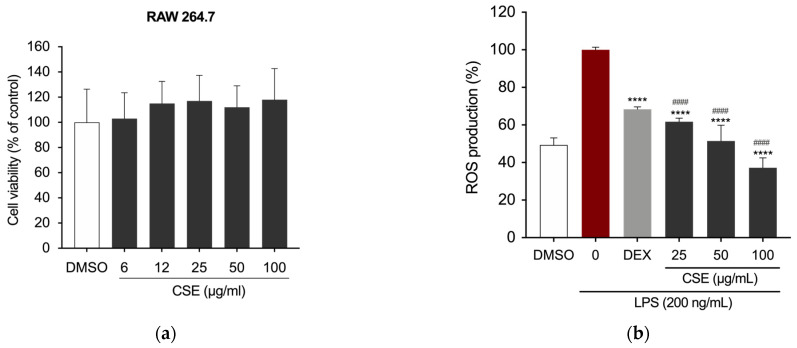
(**a**) Effects of CSE on the viability of RAW 264.7 cells. DMSO served as a vehicle. (**b**) Bar graphs depicting ROS levels measured by relative fluorescence intensity normalized to cell count using the Crystal Violet assay. **** *p* < 0.0001 (vs. LPS); #### *p* < 0.0001 (vs. DEX).

The RAW 264.7 murine macrophage cell line was employed to evaluate the anti-inflammatory potential of CSE. Initially, the extract’s potential cytotoxic effects were assessed using the MTT assay. The results in Figure 1a show cell viability, expressed as a percentage of the DMSO-treated control, with DMSO serving as the vehicle. After culturing the cells with CSE (0, 6, 12, 25, 50, and 100 μg/mL), cell viability was measured using the MTT assays after 24 h. The final concentration of DMSO did not exceed 0.1% (*v*/*v*) in both treated and untreated cells, and it did not adversely affect the analyzed parameters. None of the tested concentrations of CSE impacted RAW 264.7 cell viability (Figure 1a).

Subsequently, to evaluate the extract’s ability to mitigate the production of reactive oxygen species (ROS), intracellular ROS levels were quantified following pre-treatment with various concentrations of CSE, followed by LPS stimulation (200 ng/mL) for 5 h. Dexamethasone (DEX), a recognized anti-inflammatory drug, was used as a positive control at a concentration of 5 µg/mL. ROS levels were quantified by measuring fluorescence intensity using 2′,7′-dichlorodihydrofluorescein diacetate (DCFH_2_-DA) and normalized to cell number using Crystal Violet staining. As shown in Figure 1b, all tested concentrations significantly reduced ROS production in LPS-stimulated RAW 264.7 cells.

**Figure 2:**
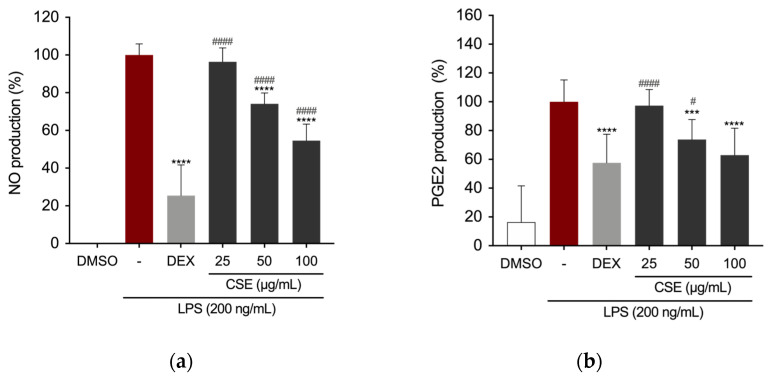

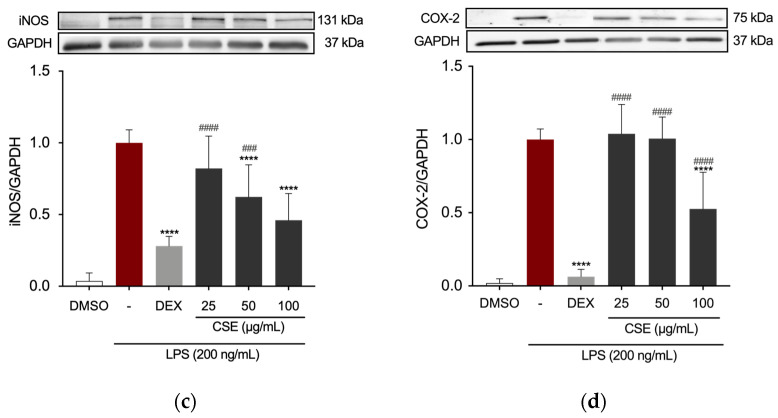
Effect of CSE on LPS-induced NO, PGE2 production, and iNOS and COX-2 protein expression levels in LPS-induced RAW264.7 cells. (**a**) NO and (**b**) PGE2 production levels measured in the supernatants of RAW 264.7 cells. Expression levels of (**c**) iNOS and (**d**) COX-2 proteins. *** *p* = 0.0001, and **** *p* < 0.0001 (vs. LPS). # *p* = 0.0378, ### *p* = 0.0004, and #### *p* < 0.0001 (vs. DEX).

The impact of CSE on reducing the production of key inflammatory mediators was assessed in RAW 264.7 cells. Cells were pre-treated with concentrations of CSE (25, 50, and 100 µg/mL) or DEX (5 µg/mL) for 4 h, followed by stimulation with 200 ng/mL LPS for 24 h. Nitric oxide (NO) production was measured using the Griess assay. DEX significantly inhibited NO production in the supernatant of RAW 264.7 cells (Figure 2a), which was also significantly reduced in the presence of CSE at 50 μg/mL and 100 μg/mL.

Similarly, cells treated with LPS in the presence of CSE at 50 and 100 μg/mL showed a notable decrease in prostaglandin E2 (PGE2) production (Figure 2b). Protein expression of iNOS and COX-2, the precursor enzymes for NO and PGE2, respectively, was assessed via Western blotting. Figure 2c illustrates a dose-dependent reduction in the expression levels of these enzymes compared to the LPS-treated group. Quantitative analysis of the immunoreactive bands indicated decreased iNOS expression in RAW 264.7 cells treated with CSE extract at concentrations of 50 μg/mL and 100 μg/mL, which correlates with the NO assay findings. COX-2 expression significantly decreased at the highest concentration of the extract (Figure 2d), thus confirming the extract’s anti-inflammatory effect.

**Figure 3:**
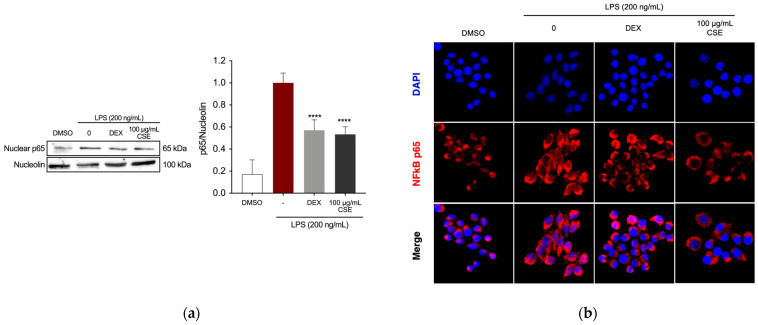
Effect of CSE on NF-κB activation in LPS-induced RAW264.7 cells. (**a**) Evaluation of NF-κB p65 expression in the nuclear compartment of RAW264.7 cells by Western blotting. **** *p* < 0.0001 (vs. LPS). (**b**) Visualization of NF-κB localization using fluorescence microscopy after staining for NF-κB p65 (red). Cell nuclei were counterstained with DAPI (blue). Images were captured at 40× magnification.

The Nuclear Factor-kappa B (NF-κB) transcription factor is crucial in mediating the production of the observed inflammatory markers by directly regulating the expression of genes encoding enzymes such as iNOS and COX-2 [49]. Therefore, we further investigated the effects of CSE by assessing the expression of the p65 subunit in the nucleus of RAW 264.7 cells following LPS induction. Following pre-treatment with DEX or CSE for 4 h, cells were then stimulated with LPS (200 ng/mL) for 1 h. Treatment with CSE at a concentration of 100 µg/mL significantly reduced the expression of NF-κB p65 in the nucleus of LPS-stimulated RAW cells (Figure 3a). Immunostaining and fluorescence microscopy analysis of NF-κB p65 localization revealed that in untreated RAW 264.7 cells, the p65 protein was predominantly localized in the cytoplasm (Figure 3b). Upon LPS stimulation, there was a notable translocation of p65 from the cytoplasm to the nucleus. However, treatment with DEX and CSE at a concentration of 100 µg/mL prevented the nuclear translocation of NF-κB, retaining it in the cytoplasm.

### 2.3. CSE Reduced IL-1β-Stimulated Production of Inflammatory Mediators in Human Chondrocytes

**Figure 4:**
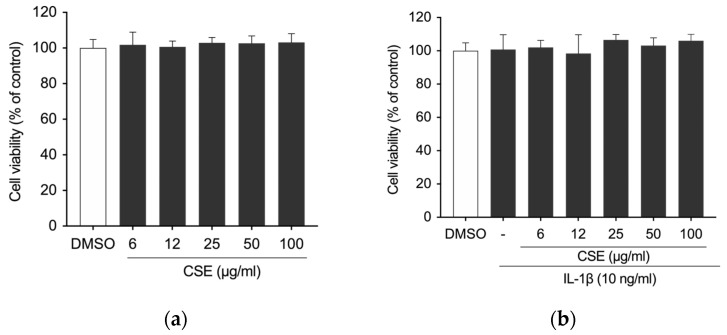
Effects of CSE on the viability of C-20/A4 cells exposed to CSE for 24 h, assessed (**a**) under basal conditions or (**b**) following IL-1β stimulation. Statistical differences were evaluated against DMSO or IL-1β. No significant differences were observed.

The potential anti-inflammatory effect of CSE was further evaluated on cells in the osteoarticular compartment using the C-20/A4 human chondrocytes cell line. The cells were pre-treated with CSE (0, 6, 12, 25, 50, and 100 μg/mL) for 4 h before being stimulated with IL-1β (10 ng/mL) for 24 h. First, possible cytotoxic effects were ruled out using the CCK-8 assay, showing no significant reduction in cell viability with CSE treatment, with or without 10 ng/mL IL-1β (Figure 4a,b).

**Figure 5:**
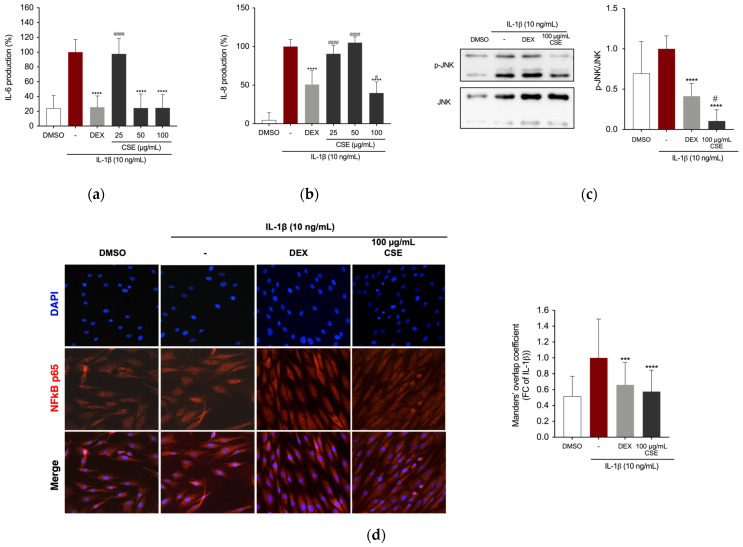
Effect of CSE on IL-1β-induced inflammatory mediators in C-20/A4 cells. (**a**) IL-6 and (**b**) IL-8 production in the supernatant of C-20/A4 cells after 24 h of stimulation. (**c**) JNK phosphorylation levels after 1 h of stimulation, measured by Western blotting. Band intensities were normalized to the non-phosphorylated form. (**d**) NF-κB p65 localization visualized after 1 h of IL-1β stimulation, stained for NF-κB (red) and DAPI (blue) at 40× magnification. The bar graph quantifies NF-κB p65 co-localization with DAPI using Manders’ coefficient. *** *p* = 0.0001 and **** *p* < 0.0001 (vs. IL-1β); # *p* ≤ 0.0438 and #### *p* < 0.0001 (vs. DEX).

After pre-treating the cells with DEX or CSE for 4 h, the cells were stimulated with 10 ng/mL IL-1β. Our findings indicate that CSE suppresses IL-6 cytokine release in the supernatant of IL-1β-stimulated cells at concentrations of 50 and 100 µg/mL (Figure 5a) and IL-8 cytokine release at a concentration of 100 µg/mL (Figure 5b). We further investigated the effects of CSE on the activation of c-Jun NH_2_-terminal kinase (JNK) using phospho-specific antibodies against this protein. IL-1β-induced phosphorylation of JNK was significantly suppressed by 100 µg/mL of CSE (Figure 5c). Immunostaining and fluorescence microscopy were used to analyze NF-κB localization in human chondrocytes. The results showed that IL-1β stimulation induced translocation of the NF-κB p65 subunit to the nucleus of C-20/A4 cells (Figure 5d). Similar to RAW 264.7 cells, treatment with DEX and CSE extract at a concentration of 100 μg/mL retained NF-κB in the cytoplasm of cells.

### 2.4. In Silico Results

#### Target/Compound Interaction

**Figure 6:**
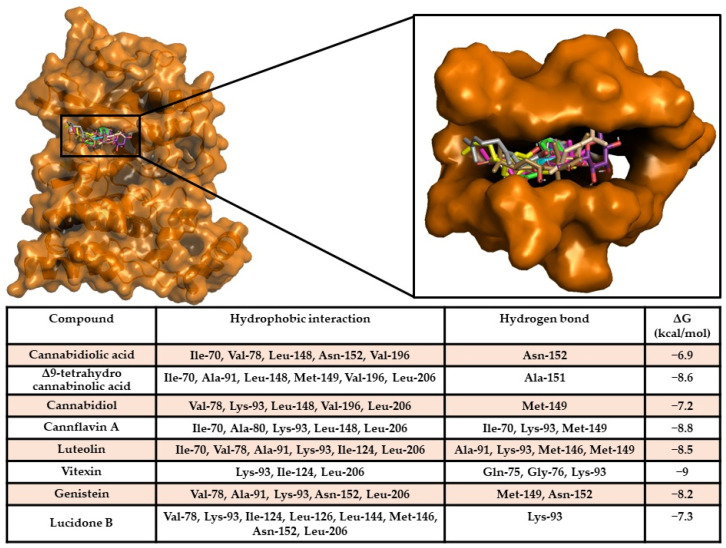
MAPK10/compound interaction overview. The 3D structure of the target is represented as an orange surface/cartoon, while the 3D structures of compounds are reported in coloured sticks. The enlargement shows the docking pose of compounds within the target binding pocket. In the table, the interaction network and binding free energy of each compound against the target are shown.

A docking simulation of compounds against MAPK10 and NF-κB p65 was performed, allowing the compounds to bind in a known binding region of human MAPK10 inhibitors [50]. The docking results provided the evidence that all compounds bound in the same binding pocket, sharing a similar binding pose. Furthermore, all compounds showed a high binding affinity of compounds on the target, revealing the ability of the compounds to spontaneously bind against the target. Interaction analyses detected a large hydrophobic and polar interaction network within a sensing binding region of the target [50] (Figure 6).

**Figure 7:**
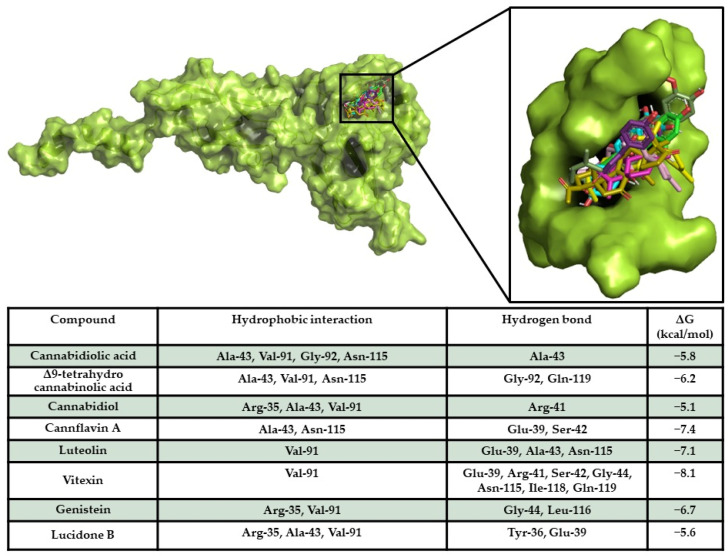
NF-κB p65/compound interaction overview. The 3D structure of the target is represented as a green surface/cartoon, while the 3D structures of compounds are reported as coloured sticks. The enlargement shows the docking pose of compounds within the target binding pocket. In the table, the interaction network and binding free energy of each compound against the target are shown.

NF-κB p65/compound docking results showed the ability of vitexin (−8.1 kcal/mol), cannflavin A (−7.4 kcal/mol), luteolin (−7.1 kcal/mol), and genistein (−6.7 kcal/mol) to strongly interact with the target with high energy scores and forming a wide hydrophobic and polar interaction network. Interestingly, all compounds docked in a target binding pocket proposed to be an inhibitor binding site of NF-κB p65 [51] (Figure 7), triggering strong polar interactions with protein sensing residues, suggesting the potential inhibitory activity of compounds on the target.

### 2.5. A HA-Based CSE Formulation Reduced LPS-Induced Inflammation in RAW 264.7 Cells

**Figure 8:**
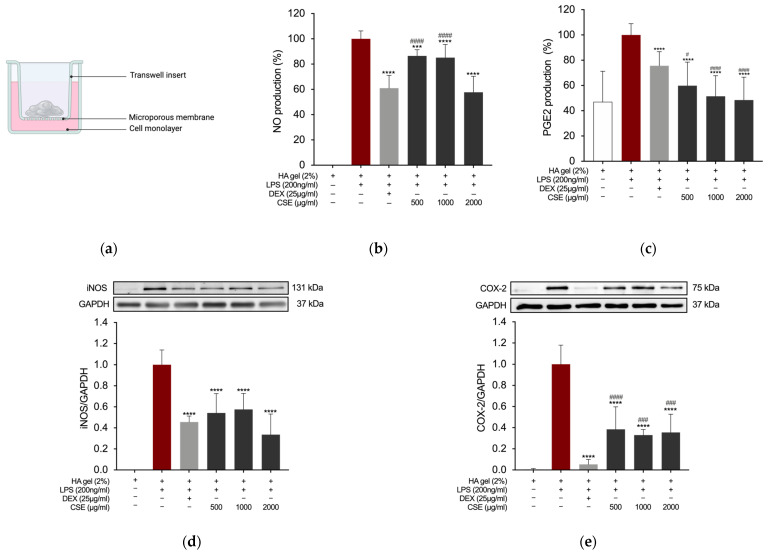
(**a**) Schematic representation of HA-based formulation vehiculation to the RAW 264.7 cells monolayer. (**b**) Effects of HA-based gel formulations containing CSE on LPS-induced NO and (**c**) PGE2 production in RAW264.7 cells. (**d**) Effects on iNOS and (**e**) COX-2 expression level. *** *p* = 0.0003 and **** *p* < 0.0001 (vs. LPS); # *p* = 0.0118, ### *p* ≤ 0.0009, and #### *p* < 0.0001 (vs. DEX).

Based on findings demonstrating a significant reduction in inflammatory markers in both LPS-stimulated RAW 264.7 murine macrophages and C-20/A4 human immortalized chondrocyte cells treated with CSE, this investigation explored whether incorporating the extract into a hyaluronic acid (HA)-based gel formulation could replicate these effects. The Griess assay quantified NO production in RAW 264.7 cells treated with the HA-based CSE (HA-CSE) formulation using Transwell inserts in 6-well plates (Figure 8a). After 24 h of exposure to HA-based formulations, cells were stimulated with LPS (200 ng/mL) for an additional 24 h. NO and PGE2 levels significantly decreased following treatment with both HA-DEX (25 μg/mL) as a positive control and HA-CSE (500, 1000, and 2000 µg/mL) across all tested concentrations (Figure 8b,c). Western blot analysis was used to evaluate iNOS and COX-2 expression in LPS-stimulated RAW 264.7 macrophages treated with HA-CSE. Accordingly, the HA-CSE formulation demonstrated robust anti-inflammatory effects on LPS-induced macrophages at the protein level (Figure 8d,e).

### 2.6. Mutagenicity Assay: Ames Test

**Figure 9:**
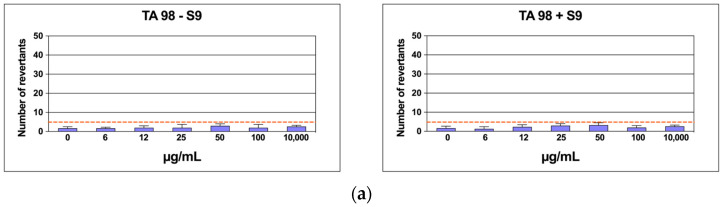

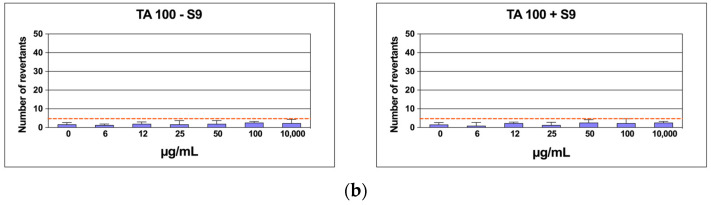
Number of revertants in TA98 (**a**) and TA100 (**b**) *S. typhimurium* strain treated with increasing concentrations of CSE with and without S9 fraction. The results are reported as the mean of revertants ± SD; *n* = 6; *p* ≤ 0.01.

To rule out any possible mutagenic effect of CSE, six concentrations of the extract were assessed on TA98 and TA100 bacteria in the *Salmonella* mutagenicity assay, both with and without S9 metabolic activation. The results in Figure 9a,b show that CSE did not exhibit genotoxicity towards TA98 and TA100 at any concentration tested, whether S9 activation was present or not. Specifically, the number of revertants was consistently lower and significantly different from the positive control up to 10,000 µg/mL (*p* ≤ 0.01). In each case, both the background levels and the positive control levels were within the typical range observed in our laboratory.

## 3. Discussion

The integration of circular economy principles with the bioeconomy, known as the circular bioeconomy, is gaining prominence across academic, political, and industrial spheres [52]. This model emphasizes the sustainable use of renewable biological resources from land and sea to produce energy, industrial goods, food, and feed, while reimagining waste as a valuable resource [53]. By optimizing resource use and reintegrating materials into production cycles, the circular bioeconomy fosters sustainable innovation, reduces environmental impacts, and supports biodiversity [54,55,56,57,58,59,60]. It bridges biotechnology, economics, and societal needs, addressing critical challenges such as resource-efficient products, biomaterials, and bioenergy production. Additionally, it aligns with global goals like the European Green Deal by facilitating carbon reintegration into natural cycles and enhancing environmental protection. Italy has emerged as a leader in this field, particularly in bio-based chemistry and compostable bioplastics, underpinned by its 2017 National Bioeconomy Strategy. The country has demonstrated how fostering collaboration between industry, agriculture, and research can drive sustainable progress [61].

One compelling application of circular bioeconomy principles lies in the utilization of residual biomass from *C. sativa*, particularly hemp. This plant, domesticated over 12,000 years ago, has been cultivated for two primary purposes: fiber and seed production (hemp) and the cannabinoid-rich female flowers used for psychoactive and medicinal purposes (drug-type “weed” *Cannabis*) [62]. Over centuries, selective breeding has led to significant genetic and phenotypic divergence between these two types. Hemp, a tall-growing variety of *C. sativa*, was traditionally bred for fiber production and is characterized by high concentrations of cannabidiolic acid (CBDA) with minimal Δ^9^-tetrahydrocannabinolic acid (THCA) content. In contrast, drug-type *Cannabis* was selected for recreational and ceremonial use, owing to its high levels of the acid precursor to psychoactive THC and its prolific production of female flowers. Recently, increased interest in CBDA-rich drug-type chemovars for pharmaceutical applications has led to the re-introduction of hemp traits into drug-type cultivars [63]. The term “chemovar” refers to *Cannabis* germplasm classified as THCA-dominant, CBDA-dominant, or balanced (with approximately equal levels of THCA and CBDA), further differentiated by their terpenoid profiles. Decarboxylation transforms these acids into their pharmacologically active forms, CBD and THC, which have distinct applications in medicine and recreation. CBD, in particular, is valued for its anti-inflammatory and anti-convulsant properties [62].

Hemp cultivation prioritizes vegetative growth and CBDA production, a trend reinforced by modern regulations limiting THC content in hemp products. It is generally grown in outdoor, low-input systems, making it environmentally and economically sustainable. By contrast, drug-type *Cannabis*, bred for high THCA yields and dense flower production, requires high-input, controlled environments to meet pharmaceutical quality standards. This divergence in cultivation practices underscores the different challenges and opportunities presented by each type [64,65]. The substantial residual biomass produced by hemp cultivation, particularly the leaves, often goes to waste and is frequently burned, contributing to air pollution [66]. However, this biomass presents a valuable opportunity within the circular bioeconomy framework for sustainable utilization. Although traditionally underutilized, this biomass could become a valuable resource for pharmaceutical cannabinoid production. The abundance of hemp by-products, combined with their low THC content, makes them an ideal candidate for cost-effective and sustainable CBD extraction. Additionally, this approach reduces reliance on drug-type *Cannabis*, which requires resource-intensive cultivation. Hemp’s adaptability to diverse environments and its lower input requirements makes it a more sustainable option. By repurposing its by-products for drug production, hemp farming can minimize waste, enhance economic viability, and support the circular bioeconomy’s goals.

In this study, the objective was to extract the secondary metabolites, in particular cannabinoids and flavonoids, which have displayed anti-inflammatory and antioxidant properties [17,27,28], from the leaves of *C. sativa* to repurpose the waste material. The cultivar used was “Futura 75”, a monoic, multi-purpose variety selected in France for seed, CBD, and biomass production. Its complex chemical composition, characterized by a rich array of secondary metabolites [67], presents significant challenges for the complete extraction of all the components. The selection of an appropriate extraction method is crucial for obtaining both high quantity and quality of the extracted molecules. The GRAS (Generically Recognized As Safe) [68] ethanol–water (70:30 *v*/*v*) mixture was chosen as the extraction solvent for this study. Conventional techniques for extracting flavonoids from plant matrices typically involve various solvent combinations such as water, ethanol, methanol, acetone, and ethyl acetate, either individually or in combination. These methods often yield high extraction efficiencies of compounds with antioxidant properties [69]. The results from evaluating the TPC and TFC of CSE confirmed its effectiveness in extracting antioxidants from *C. sativa* leaves using the hydroethanolic mixture. Ahmed et al. reported TPC values for ethanolic, methanolic, and aqueous extracts of *C. sativa* leaves of 2.70 ± 0.109 mg GAE/g, 36.42 ± 1.905 mg GAE/g, and 29.98 ± 0.56 mg GAE/g, respectively [70]. Luangpraditkun et al. reported a TPC of 4 mg GAE/g dry extract in hemp leaf extract [71]. The calculated TPC for CSE was substantially higher, representing 65.31 ± 2.83 mg GAE/g dry extract, corresponding to 8.5 mg GAE/g dry weight, which aligns with Chatzimitakos et al., who reported a TPC for *C. sativa* hemp leaf water extracts ranging from 7.28 to 9.71 mg GAE/g dry weight [72]. Mkpenie et al. reported TPC concentrations ranging from 0.09 to 0.556 mg GAE/g of dried *C. sativa* leaves using various solvents and extraction methods [73]. Furthermore, the TFC values in the hydroethanolic extract (55.18 ± 10.6 mg QE/g dry extract) were similar to those reported by Ahmed et al. for methanolic and ethanolic extracts (59.03 ± 1.31 mg QE/g and 56.00 ± 1.85 mg QE/g, respectively), followed by hexane (17.35 ± 0.43 mg QE/g) and acetone (12.08 ± 0.62 mg QE/g) [2]. Luangpraditkun et al. reported an EC50 for DPPH radical scavenging of 277.9 ± 2.41 µg/mL [71], much higher than the IC50 calculated for CSE, which was 67.93 ± 3.6 µg/mL. Overall, CSE exhibited significant amounts of phenolic compounds, contributing to its notable antioxidant capacity as assessed by the potassium ferricyanide, ABTS, and DPPH methods. Particularly noteworthy is the IC50 value of CSE on the ABTS radical, similar to that of Trolox standard compound, indicating its potent radical scavenging activity.

UPLC-MS/MS profiling confirmed the presence of flavonoids, which were the second most abundant class of secondary metabolites found in CSE, and among them, the predominant were cannflavin A, luteolin, vitexin, genistein and lucidone B. Nevertheless, the main class of secondary metabolites found in CSE was cannabinoids. Comparing the composition of our extract with other relevant studies reveals notable differences in the relative abundance of key compounds, underlining how cultivar selection, cultivation conditions, and extraction methodologies can significantly shape chemical profiles. CBDA is the dominant compound in our extract, comprising 78.92% of the total composition, while Chen et al. report a lower proportion, generally within the 40–50% range [74]. This suggests that our extract may originate from a cultivar specifically optimized for CBDA production or an extraction process that prioritizes this compound. This CBDA-rich profile is significant for applications targeting its pharmacological potential, such as anti-inflammatory and anti-nociceptive effects.

Interestingly, THCA accounts for 11.91% of our extract, a higher percentage compared to the <5% levels typically reported in Chen et al.’s work [74]. In contrast, CBD, the decarboxylated form of CBDA, represents only 3.56% of our extract, compared to the 10–15% levels noted by Chen et al. This difference suggests that our extract underwent minimal decarboxylation, likely preserving acidic cannabinoids like CBDA and THCA. Such preservation could reflect intentional processing methods, as acidic cannabinoids are increasingly recognized for their distinct therapeutic properties. Similarly, Luangpraditkun et al., in a study of hemp leaf extracts obtained using reflux ethanol methods, reported higher CBD levels compared to THC [71], while Jin et al. observed substantial amounts of CBD, CBDA, and THC in methanol–based extracts. These findings highlight the influence of extraction techniques on cannabinoid profiles and underscore the distinct chemical characteristics of our extract [75].

Flavonoids also differ substantially between studies. Cannflavin A, a flavonoid with notable anti-inflammatory properties, is present at 1.45% in our extract, significantly higher than the <0.5% levels reported by Chen et al. [74]. This suggests a broader spectrum of secondary metabolites in our extract, potentially due to cultivar-specific traits or an extraction technique that enhances flavonoid recovery. Similarly, luteolin is relatively abundant in our extract at 1.09%, compared to trace levels in Chen et al.’s work. Notably, Chatzimitakos et al. also detected luteolin in the water extract of hemp leaves [72], emphasizing its potential enrichment in specific extraction methods. Vitexin, another flavonoid detected at 0.63% in our extract but nearly absent in Chen et al.’s findings, aligns with Jin et al.’s report of its presence in an acid hydrolyzation extract of *C. sativa* leaves [75]. These flavonoids enhance the pharmacological potential of our extract, offering antioxidant and anti-inflammatory benefits.

Our extract also includes genistein at 0.57%, a compound rarely reported in *C. sativa* studies, highlighting its unique isoflavonoid content. This compound may contribute antioxidant and estrogenic properties, which could broaden the extract’s therapeutic applications. Additionally, lucidone B, present at 0.20% in our extract and absent in Chen et al.’s work, points to distinct metabolic pathways in the cultivar used or higher sensitivity in our analytical methods.

Overall, our extract demonstrates a strong focus on CBDA and a richer diversity of secondary metabolites compared to Chen et al. and other studies. This composition suggests potential pharmacological advantages, particularly where a diverse profile of cannabinoids and flavonoids is desirable. However, the relatively higher THCA content in our extract may require careful consideration in regulatory contexts, depending on the intended applications. This analysis underscores the critical role of cultivar selection and extraction methodologies in shaping *C. sativa* extracts, ultimately influencing their pharmacological utility and regulatory compliance. The versatility of CBD’s pharmacology is well known, while the potential benefits of its precursor CBDA are not fully exploited. CBD has been demonstrated to exhibit modulatory effects on THC activity at CB1 receptors. Additionally, it possesses analgesic and anti-inflammatory properties and serves as a potent neuroprotective antioxidant [30,76]. The bioactivity of CBD is vast, and its pharmacological potential has not been fully explored due to the complex history of the source plant. It has been suggested for the management of inflammation and joint pain [38,41,77] and is an antagonist of TNF-α in animal models affected by rheumatoid arthritis [32,44,45]. CBDA is the acidic precursor of CBD, which is decarboxylated by heating, consequently producing free CBD [78]. CBDA has been reported to inhibit COX-2 activity [76]. It has been suggested that the carboxylic acid moiety in CBDA is a key determinant for the inhibition and that naturally occurring CBDA in *C. sativa* is a selective inhibitor for COX-2 [28]. CBDA is a prominent secondary metabolite in *C. sativa* by-products. It is a major cannabinoid in hemp seed oil and serves as a marker for storage conditions and production processes [76]. The present study showed that *C. sativa* leaves are rich in such an interesting compound and that an easy and sustainable method, such as the hydroethanolic extraction from *C. sativa* leaves employed in this study, could yield a high percentage of CBDA, as indicated by UPLC-MS/MS analysis, making it a promising development.

In light of these findings, we examined the potential anti-inflammatory effects of hydroethanolic *C. sativa* leaves on RAW 264.7 macrophages and IL-1β- stimulated human immortalized chondrocytes. Inflammation plays a crucial role in the development of several chronic non-communicable diseases, including OA. Currently, no effective treatments are available to slow down the progression of OA, despite the use of several pharmaceutical drugs, and long-term side effects are reported in older, frail, and comorbid patients. Consequently, plant-derived agents with low toxicity and anti-inflammatory activity are increasingly being utilized as a therapeutic alternative for OA treatment [37,38,79,80,81].

During the progression of OA, pro-inflammatory cytokines like TNF-α and IL-1 become activated, leading to inflammation and the destruction of cartilage [82]. However, commonly used pharmacological treatments like oral non-steroidal anti-inflammatory drugs have serious adverse effects [83]. Chondrocytes in OA patients are stimulated by external stress, leading to the upregulation of catabolic molecules, including COX-2 [82]. Inflammatory cytokines upregulate COX-2 expression, which promotes the degradation of proteoglycan and collagen, leading to chondrocyte apoptosis [84]. Additionally, increased inflammation in osteoarthritis upregulates the release of destructive mediators such as NO and prostaglandins, via the regulation of iNOS expression and generation of ROS in chondrocytes [80]. Their involvement is also demonstrated by the elevated levels of PGE2 and collagenase in osteoarthritic patients, promoting the degradation of collagen in cartilage [79]. In our study, we observed a concentration-dependent decrease in LPS-mediated secretion of PGE2 and NO production, as well as a reduction in the expression of their progenitor enzymes COX-2 and iNOS in stimulated RAW 264.7 cells after treatment with CSE. The analysis of nuclear expression of the p65 subunit of NF-κB transcription factor and immunofluorescence studies revealed that pre-treatment with CSE at the highest concentration tested led to the retention of NF-κB p65 in the nucleus after LPS stimulation, suggesting that CSE may exert its anti-inflammatory effects via the NF-κB pathway. These findings are promising as NF-κB signaling plays a critical role in OA progression, and blocking its activity could be a therapeutic advantage [81].

Studies have shown that methanolic extract obtained from *C. sativa* leaves can decrease NO production in a dose-dependent manner in IL-1β-stimulated SW932 synovial cells, as reported by Duangnin et al. [85]. IL-1β is a pro-inflammatory cytokine that plays a crucial role in the pathogenesis of OA and is released by activated synoviocytes or chondrocytes. Several studies have revealed that IL-1β induces NO production by stimulating iNOS activity and increases the expression of PGE2 via COX-2 activation. Moreover, IL-1β upregulates the expression of other pro-inflammatory cytokines, such as IL-6, and chemokines, including IL-8, leading to matrix remodeling, which eventually leads to the degradation of vital matrix constituents [82,86]. Therefore, IL-1β-stimulated chondrocytes are widely used as an in vitro model of OA [79,81,86,87]. Elevated levels of IL-6 were found in C-20/A4 human immortalized chondrocyte cells stimulated with IL-1β. Treatment with CSE markedly reduced IL-6 and IL-8 levels in the supernatant of stimulated cells. Additionally, CSE blocked the activation of JNK MAPK. Scientific studies demonstrate that JNK activation in OA triggers cartilage degradation by phosphorylating c-Jun, thereby reducing proteoglycan synthesis and increasing MMP-13 production, making the JNK pathway a promising target for OA therapy [88,89]. NF-κB p65 localization visualized upon IL-1β stimulation and quantified using Manders’ coefficient also confirmed the involvement of the NF-κB pathway. This is particularly relevant as several IL-1β-induced inflammatory mediators in chondrocytes are regulated by the NF-κB signaling pathway [82,86,90], which includes pro-inflammatory cytokines, adhesion molecules, and proteases such as PGE2, NO, and IL-6 [90].

Studies have suggested that flavonoid dietary intake is inversely associated with age-related diseases such as cardiovascular diseases, neurodegeneration, and type 2 diabetes [91,92,93]. The anti-inflammatory activity was linked to their ability to modulate the expression of molecules such as pro-inflammatory enzymes including COX-2, iNOS, and pro-inflammatory cytokines such as IL-1 and IL-6 [94]. Cannflavin A and cannflavin B, prenylated flavonoids unique to *C. sativa*, have shown significant anti-inflammatory effects [46,95]. In the earliest studies on these compounds, Barrett and colleagues were the first to identify cannflavins as the active agents responsible for inhibiting PGE2 production in human rheumatoid synovial cells treated with *C. sativa* extracts. These compounds exhibited anti-inflammatory effects estimated to be approximately thirty times more potent than aspirin [96,97]. Later research demonstrated that the potent anti-inflammatory properties of cannflavin A and B are due to their ability to inhibit microsomal prostaglandin E2 synthase (mPGES)-1. This finding is particularly interesting because this enzyme is responsible for the significant formation of PGE2 during inflammation. Identifying inhibitors for mPGES-1 could offer a safe alternative to non-steroidal anti-inflammatory drugs [95,98]. Lim et al. reported that luteolin and genistein, which were among other flavonoids identified in CSE, effectively reduced elevated levels of inflammatory cytokines such as IL-1β and IL-6, as well as NF-κB activation in aged animal models [94]. Luteolin has exhibited significant anti-inflammatory properties by reducing the production of various pro-inflammatory agents, including NO, PGE2, tumor necrosis factor-alpha (TNF-α), matrix metalloproteinase-2 (MMP-2), matrix metalloproteinase-8 (MMP-8), and matrix metalloproteinase-9 (MMP-9). Furthermore, luteolin has been shown to downregulate the expression of COX-2, iNOS, matrix metalloproteinase-3 (MMP-3), and matrix metalloproteinase-13 (MMP-13). These effects have been observed in both in vitro and in vivo models of arthritis, highlighting luteolin’s potential as an effective anti-inflammatory agent [6]. Previous studies have shown that vitexin significantly reduces inflammatory mediators by modulating the MAPK/NF-κB signaling pathways [99]. Notably, in OA models, vitexin inhibited MMPs and the HIF-1α pathway in IL-1β-stimulated OA chondrocytes, reducing pro-inflammatory cytokines (IL-6, TNF-α) and enhancing cell survival. It also suppressed NO and PGE2 production in chondrocyte cultures, alleviating inflammatory injuries in OA patient-derived chondrocytes through inhibition of HIF-1α expression [100]. Furthermore, an in vivo study using a collagen-induced arthritis (CIA) rat model demonstrated vitexin’s anti-inflammatory effects by reducing inflammatory markers and cytokines (IL-1β, IL-6, IL-17, TNF-α) and modulating the JAK/STAT/SOCS signaling pathway. Vitexin normalized aberrant inflammatory mediator levels and improved histological changes in arthritic joints, further supporting its potential in rheumatoid arthritis treatment [101]. All these effects, combined with the previously mentioned anti-inflammatory properties of cannabinoids, strongly reinforce the potential of our extract for therapeutic applications. Moreover, in silico docking showed the ability of compounds to spontaneously bind in a sensing region of MAPK and NF-κB, triggering a strong interaction network with the binding residues, suggesting their potential inhibitory activity against the targets, supporting in vitro evidence.

Overall, our findings suggest that CSE has protective effects on inflammation without observed cytotoxic or mutagenic effects, achieved by reducing inflammation through the inhibition of MAPK and NF-κB signaling pathways. Subsequently, our focus shifted towards evaluating its therapeutic potential for treating inflammation-related conditions of the osteoarticular system. We investigated the feasibility of incorporating our extract into an HA-based gel formulation, leveraging HA gel as an optimal carrier for delivering anti-inflammatory medications to joints due to its viscosity, which mirrors the role of natural HA in joint function [102]. The potential of HA-CSE to modulate NO and PGE2 production and the expression of iNOS and COX-2 was investigated by testing the effect of an HA gel combined with CSE on LPS-stimulated macrophages, similar to the study conducted with CSE extract alone. This study’s findings demonstrate that incorporating the extract from *C. sativa* leaves into an HA-based formulation holds biotechnological promise.

Delivering *C. sativa* extracts to arthritic joints through HA-based formulations could be a novel approach, merging the mechanical benefits of viscosupplementation of HA with the anti-inflammatory properties of plant-derived extracts [103]. In the case of CSE, which is rich in cannabinoids and flavonoids with promising anti-arthritic effects, this dual-action strategy holds great potential for managing joint inflammation and degeneration. However, the approach is not without its challenges. CSE, as a crude extract, has variability in its composition and bioactive content, complicating standardization and dosing. Additionally, cannabinoids are inherently hydrophobic and prone to degradation in gastric and hepatic environments, resulting in low bioavailability [104].

Advanced delivery technologies are crucial for overcoming these barriers. HA-based formulations provide an alternative that addresses many of these challenges by enabling localized and sustained delivery of cannabinoids directly to joint spaces. HA is widely recognized for its biocompatibility and its utility in intra-articular (IA) injections, where it restores the viscoelastic properties of synovial fluid and provides structural support to damaged joints [105]. Evidence from HA-alendronate (HA-ALD) models has demonstrated the ability of such systems to target inflammation and protect cartilage by reducing collagen release and matrix metalloproteinase-13 (MMP-13) expression [105]. Integrating CSE into HA matrices leverages these benefits while enabling the effective delivery of hydrophobic cannabinoids to arthritic joints. Advanced HA-based systems, such as HA conjugates and ternary complexes with cyclodextrins and vitamin E (HCV systems), have demonstrated significant potential for enhancing the delivery of hydrophobic drugs while preserving the antioxidant properties of their components. [103]. By localizing drugs directly within the joint space, these systems minimize systemic degradation and ensure sustained therapeutic activity, thereby reducing the frequency of dosing required.

Comparative studies on cannabinoid carriers, such as *Rapae oleum* and Cremophor, further underscore the importance of selecting appropriate excipients to optimize bioavailability [106]. Nanotechnology-based encapsulation methods within HA matrices offer pathways for achieving localized, sustained delivery while reducing systemic side effects and maximizing therapeutic efficacy. These multifunctional HA systems not only provide effective drug delivery but also offer additional benefits, such as antioxidant properties, that enhance overall therapeutic outcomes.

In summary, the integration of CSE into HA-based formulations represents a transformative approach to managing arthritic inflammation. By combining HA’s mechanical support with the anti-inflammatory properties of cannabinoids, this strategy can address many of the limitations associated with traditional cannabinoid delivery methods. Continued advancements in HA-based drug delivery systems promise to refine this approach further, paving the way for a localized, sustained, and patient-friendly therapy for joint diseases such as osteoarthritis.

The findings of this study are promising, warranting further investigations to explore the molecular mechanisms underlying the effects of CSE. Additionally, studies focusing on its bioavailability will be essential to fully understand and optimize its therapeutic potential. Since currently available OA drug treatments only alleviate symptoms but do not reverse or cure the progression of the disease, the discovery of compounds able to prevent or inhibit the onset of OA progression would be significant. Furthermore, the fact that such a medicament could be derived from a waste product produced annually in increasing amounts is even more significant in terms of biomass sustainability and reducing environmental impact.

## 4. Materials and Methods

### 4.1. Materials

Dulbecco’s Modified Eagle’s Medium (DMEM), trypsin solution, and all the solvents used for cell culture were purchased from Merck (Darmstadt, Germany). RAW 264.7 cells were from the American Type Culture Collection (Manassas, VA, USA). An Ames test kit was supplied from Xenometrix (Allschwil, Switzerland).

### 4.2. Preparation of C. sativa Hydroethanolic (CSE) Extract

In the third decade of April 2021, the crop was sowed on loam soil located 20 km N.E. from Siena (Italy). The cultivar used was Futura 75, a monoic, multi-purpose variety selected in France for seed, CBD, and biomass production. The crop was harvested in the last decade of August. Leaves were hand-separated from stems, stored in a vacuum-packed bag at 4 °C, and then delivered to the lab for analysis. Following manual cleaning to remove visible residues, such as dust or other undesirable material, the leaves were oven-dried in a drying cabinet (KW.86/AV, KW Apparecchi Scientifici, Siena, Italy) at 40 °C until they reached a constant weight. It was then ground using a laboratory mixer (Microtron™ MB 550, Kinematica™) and sieved to a fine powder (Sigma-Aldrich analytical sieve Z289744-1EA, pore size 250 μm, Sigma Aldrich, St. Louis, MO, USA). Pulverized leaves were extracted by heath-reflux extraction with an ethanol–water (70:30 *v*/*v*) mixture using a sample/solvent ratio equal to 1:10 (g/mL), for three hours at 80 °C. The supernatant was then separated from the residual biomass, filtered, and subjected to rotary evaporation to remove the organic solvent. Finally, the aqueous residue was freeze-dried to produce 1.31 ± 0.56 g (or 13.1% *w*/*w*) of dry extract. The extraction was carried out in duplicate. Following extraction, 100 mg of dry extract was dissolved in 1 mL of 100% DMSO to obtain a 100 mg/mL CSE stock solution. This solution was then aliquoted and stored in the refrigerator at −32 °C for subsequent analyses.

### 4.3. Total Phenolic Content (TPC)

Total TPC was quantified by the Folin–Ciocalteu (FC) method [107] with some modifications. A calibration curve was generated using gallic acid (GA) solutions in the concentration range of 20–120 μg/mL. CSE samples were prepared by diluting the stock solution (1 mg/mL) in milli-Q water. Standard and sample tubes were then mixed with 1 mL of 1N FC reagent in milli-Q water. After 3 min, 1 mL of saturated Na_2_CO_3_ and 7 mL of milli-Q water were added. All tubes were incubated for 90 min at room temperature, shielded from light, before measuring absorbance at 725 nm. Simultaneously, a solution containing all reagents with the extract solvent alone were prepared as blank. TPC was expressed as milligrams of GA equivalent (GAE) per gram of dry extract.

### 4.4. Total Flavonoid Content (TFC)

The determination of the TFC was carried out according to the aluminum chloride (AlCl_3_) colorimetric method [108]. A calibration curve was generated using quercetin (Q) solutions in the concentration range of 20–200 μg/mL. CSE samples were prepared by diluting the stock solution (1 mg/mL) in milli-Q water. A total of 500 μL of standard/sample were mixed to 100 μL of 10% AlCl_3_ in 1 M potassium acetate and 3.3 mL of ethanol. Each solution was prepared in triplicate. After 30 min of incubation, the absorbance was measured at 430 nm using an EnVision system (PerkinElmer, Waltham, MA, USA). The results were expressed as mg of Q equivalent (QE) per gram of extract.

### 4.5. Determination of Reducing Power

The reducing power (RP) of CSE was assessed using the potassium ferricyanide reducing power assay, following a modified version of the method described by [109]. A calibration curve was generated using ascorbic acid (AA) solutions in the concentration range of 20–140 μg/mL. CSE samples were prepared by diluting the stock solution (1 mg/mL) in milli-Q water. A blank was created with water.

The samples, standards, and blank were treated with 1 mL of 0.2 M phosphate buffer (K_2_HPO_4_:KH_2_PO_4_) at pH 6.6 and 1 mL of 1% potassium ferricyanide (K_3_[Fe(CN)_6_]), followed by incubation at 50 °C for 20 min. Subsequently, 1 mL of 10% (*w*/*v*) trichloroacetic acid was added to each solution, allowing an additional incubation at room temperature for 10 min. After this step, 2.5 mL of milli-Q water and 0.5 mL of 0.1% (*w*/*v*) ferric chloride (FeCl_3_) solution were added to 2.5 mL of the mixture before measuring the absorbance at 700 nm. The antioxidant power was quantified as mg AA equivalents (AAE) per gram of dry extract.

### 4.6. ABTS^•+^ Free-Radical Scavenging Activity

The Trolox equivalent antioxidant capacity (TEAC) assay is based on the conversion of oxidized ABTS^•+^ radicals to ABTS by molecules able to neutralize the radical [110]. The assay was performed using the OxiSelect™ TEAC Assay Kit (Cell Biolabs Inc., San Diego, CA, USA) according to the manufacturer’s instructions. Briefly, 25 µL of different concentrations of sample were added to 150 µL of freshly prepared ABTS^•+^ reagent diluted 1:50 in the appropriate diluent in a 96-well plate. After 5 min incubation on an orbital shaker, the absorbance was measured at 405 nm. Results were expressed as IC50 (µg/mL) (i.e., inhibitory concentration causing a 50% decrease in absorbance).

### 4.7. DPPH Free-Radical Scavenging Activity

DPPH free-radical scavenging activity was estimated by dosing the free DPPH (2,2-diphenyl-1-picrylhydrazyl) radical according to the method of Yen and Chen [111], with some modifications [112]. Briefly, 100 µM of DPPH was added to each standard/sample dilution (both in the concentration range of 5–100 µg/mL), and the solutions were incubated 30′ in the dark, at 37 °C. The reaction was monitored at 517 nm to determine the percentage of discoloration. Τrolox (T) was used to set the standard curve. The capability to scavenge the DPPH radical was reported as IC50 (µg/mL) (i.e., inhibitory concentration causing a 50% decrease in absorbance).

### 4.8. UPLC-MS/MS

To investigate the non-volatile profile of CSE extract, an Ultimate 3000 UPLC system (Thermo Fisher Scientific, Waltham, MA, USA) was used that was controlled with Thermo Xcalibur software version 4.3.73.11 (Thermo Fisher Scientific, Waltham, MA, USA). The dry CSE extract was dissolved in the ethanol–water (70:30 *v*/*v*) mixture before injection into the UPLC-Q-Exactive Plus system for analysis, as detailed in a previous study [113].

### 4.9. Cell Cultures

RAW 264.7 [ATCC (Manassas, VA, USA)] and C-20/A4 (Sigma-Aldrich, St. Louis, MO, USA, SCC041) cells were cultured in Dulbecco’s Modified Eagle’s Medium (DMEM) containing 10% *v*/*v* FBS, 100 mg/mL penicillin, and 100 mg/mL streptomycin. Cultures were maintained at 37 °C in a humidified atmosphere of 5% CO_2_. Comparative analysis was performed with cell populations at the same generation.

#### 4.9.1. Cell Viability

RAW 264.7 cells were seeded at a density of 1 × 10^4^ cells/well in 96-well plates and cultured until sub-confluence (80–85% confluence). Cells were treated with different concentrations (6, 12, 25, 50, and 100 µg/mL) of CSE prepared in DMSO (Sigma-Aldrich) and diluted in medium; the final DMSO concentration was kept below 0.1% *v*/*v* throughout the experiment. The control was treated with DMSO at a concentration of 0.1% *v*/*v*, corresponding to the highest concentration tested. After 24 h of treatment, cells were washed with sterile PBS and MTT was added to a final concentration of 1 mg/mL. After a 2 h incubation, cells were lysed with 150 µL of DMSO. The absorbance was measured at 550 nm using an EnVision system (PerkinElmer, Waltham, MA, USA) and the percentage of cell viability was calculated relative to the control.

The viability of the C-20/A4 human immortalized chondrocyte cell line was measured using the Cell Counting Kit-8 (CCK-8) (Sigma-Aldrich, USA) according to the manufacturer’s instructions. Briefly, C-20/A4 cells were seeded at 5 × 10^3^ cells/well, in 96-well plates. After 24 h, the cells were treated with different concentrations (6; 12, 25, 50, and 100 µg/mL) of CSE prepared as before, with or without the presence of 10 ng/mL IL-1β. After 24 h of treatment, cell viability was measured at 450 nm using a microplate reader (CLARIOstar, BMG Labtech, Ortenberg, Germany). The percentage of viable cells was determined relative to the vehicle control.

Data are presented as mean ± SD from three independent experiments. Statistical differences were evaluated against DMSO or IL-1β using a one-way ANOVA with Dunnett’s post hoc test.

#### 4.9.2. Cell Stimulation

RAW 264. 7 and C-20/A4 cells were treated with CSE for 4 h prior to 24 h stimulation with lipopolysaccharide (LPS) (obtained from *Escherichia coli* O111:B4, Sigma-Aldrich) or IL-1β (Sigma-Aldrich), respectively. Dexamethasone (DEX) (Sigma-Aldrich), commonly used to treat inflammation, was used as a positive control at a concentration of 5 µg/mL.

#### 4.9.3. Quantification of Intracellular ROS Formation

The generation of reactive oxygen species (ROS) within RAW 264.7 cells was determined in 96-well plates with 2′,7′-dichlorodihydrofluorescein diacetate (DCFH_2_-DA), which is intracellularly deacetylated and oxidized to highly fluorescent 2′,7′-dichlorofluorescein (DCF) [114]. After pre-treatment with different concentrations of CSE (25, 50, and 100 μg/mL), cells were stimulated with LPS (200 ng/mL) for 5 h. DCFH_2_-DA (10 µM) dissolved in HBSS was applied to the cells and incubated at 37 °C. The plate was scanned using an EnVision system (PerkinElmer) with excitation wavelength of 485 nm and emission wavelength of 535 nm. Afterwards, the number of cells in each well was determined using the Crystal Violet assay [115]. Results were normalized to the relative cell count for each well and expressed as the relative ROS production % (RFI) with respect to the LPS group [116]. *p*-values were calculated using a one-way ANOVA with Tukey’s post hoc test.

#### 4.9.4. Determination of NO Production

The production of nitric oxide (NO) in the supernatant of RAW 264.7 cells was determined in 6-well plates (1 × 10^6^ cells/well) cultured until sub-confluence (80–85%). After treatment with CSE at different concentrations (25, 50, and 100 μg/mL) for 4 h, the cells were stimulated with LPS (200 ng/mL) for 24 h. Following stimulation, 100 µL of conditioned medium from each well was transferred to a new 96-well plate and mixed with an equal volume of Griess reagent composed of 1% sulfanilamide and 0.1% N-(1-naphthyl) ethylenediamine dihydrochloride in 5% phosphoric acid. After incubation at room temperature for 10 min, the absorbance was measured at 540 nm using an EnVision system (PerkinElmer). The nitrite concentration was assessed by a sodium nitrite standard curve.

#### 4.9.5. Enzyme-Linked Immunosorbent (ELISA) Assay

RAW 264.7 and C-20/A4 cells (5 × 10^6^ cells/mL) were seeded in 6-well plates and cultured for 24 h. After treatment with CSE at different concentrations (25, 50, and 100 μg/mL) for 4 h, the cells were stimulated with LPS (200 ng/mL) or IL-1β (10 ng/mL) for 24 h. DEX (5 µg/mL) was used as a positive control. Then, the culture supernatants were collected. The concentration of PGE2 in the supernatants of RAW 264.7 cells was detected using a PGE2 ELISA kit (Cat# E-EL-0034, Elabscience, Houston, TX, USA) according to the manufacturer’s instructions. The concentration of IL-6 in the culture supernatants of C-20/A4 cells was measured using a human IL-6 (Sigma-Aldrich) ELISA Kit, according to the manufacturers’ instructions.

#### 4.9.6. Protein Extraction

Whole-cell lysates were obtained using RIPA buffer, supplemented with phosphate and protease inhibitors, and then disrupted by sonication for 15 min in an ice bath. Protein concentration was assessed using the BCA protein assay. Nuclear fractionations were obtained using the NE-PER™ Cytoplasmic and Nuclear Protein Extraction Kit (Thermo Fisher Scientific, Rockford, IL, USA) according to the manufacturer’s protocol.

#### 4.9.7. Western Blotting

A total of 20 μg of protein was resolved by SDS–PAGE and transferred onto a nitrocellulose membrane. The membrane was blocked in TBS 5% *w*/*v* nonfat dry milk at RT with gentle shaking for 2 h. The membrane was incubated with anti-iNOS (rabbit polyclonal IgG, 1:10,000 Sigma-Aldrich), anti-COX-2 (rabbit polyclonal IgG, 1:4000 Sigma-Aldrich), anti-NF-κB p65 (mouse monoclonal clone 1G10.2, 1:1000 Sigma-Aldrich), anti-nucleolin (rabbit polyclonal, 1:10,000 Sigma-Aldrich), anti-p-JNK and anti-JNK (rabbit polyclonals, 1:1000 Sigma-Aldrich), and anti-GAPDH HRP-conjugated (1:50,000) primary antibodies, ON at 4 °C. The blots were washed three times and incubated with anti-rabbit HRP-conjugated secondary antibodies (Sigma-Aldrich) 1:80,000 or anti-mouse HRP-conjugated secondary antibodies (Sigma-Aldrich) 1:50,000 for 1 h, RT. After washing three times, immunoreactive bands were detected using ECL (LuminataCrescendo, Merck Millipore, Burlington, MA, USA) and images acquired by LAS4000 (GE Healthcare, Chicago, IL, USA). The optical densities of immunoreactive bands were analyzed by ImageQuantTL software (GE Healthcare, Chicago, IL, USA, V 7.0) using GAPDH, nucleolin, or JNK as loading normalizing factors. All data are presented as mean ± SD of three independent experiments. *p*-values were calculated using a one-way ANOVA with Tukey’s post hoc test.

#### 4.9.8. Immunofluorescence Study

RAW 264.7 and C-20/A4 cells were grown on glass coverslips for 24 h; subsequently, the cells were pre-treated with CSE at 100 μg/mL for 4 h and were stimulated with LPS or IL-1β, respectively. The cells were fixed with 4% paraformaldehyde dissolved in PBS for 15 min and then permeabilized with 0.5% TritonX-100 in PBS for 5 min. After blocking, cells were incubated overnight at 4 °C with anti-NF-κB p65 (clone 1G10.2) mouse monoclonal antibodies (Sigma-Aldrich). Cells were then incubated for 1 h at room temperature with Alexa 594-conjugated goat anti-Mouse IgG (Life Technologies, Carlsbad, CA, USA). Finally, the samples were mounted with a fluoroshield mounting medium with DAPI (Abcam, Cambrige, UK). Images were captured by fluorescence microscopy (Zeiss AxioLabA1, Oberkochen, Germany). The quantitative co-localization analysis of NF-κB p65 and DAPI signals was performed using ImageJ and the JACoP plug-in to determine Manders’ coefficient [113], which represents the percentage of NF-κB p65 pixels that overlap with DAPI pixels [117]. All data are presented as mean ± SD of three independent experiments. *p*-values were calculated by a one-way ANOVA with Tukey’s post hoc test.

### 4.10. Preparation of the HA-Based CSE Formulation and RAW 264.7 Cells Treatment

The HA-based CSE formulation was produced by combining 10 mg HA powder with 500 μL of water or various concentrations (500, 1000, and 2000 μg/mL) of CSE. The mixture was placed in a glass syringe and gelified for one hour. To avoid contamination, this step was carried out in a laminar flow cabinet.

RAW 264.7 murine macrophages (4 × 10^5^ cells/well) were seeded in 6-well plates in complete DMEM at 37 °C in a humidified atmosphere at 5% CO_2_. Transwell inserts were used to test the biological activity of the formulations (500 μL/Transwell insert). A RAW 264.7 cell monolayer was grown in six different wells for: control cells, HA gel without extract, HA-DEX (25 μg/mL) as positive control, and different concentrations (500, 1000, 2000 μg/mL) of HA-CSE. Each of the formulations was placed on a different Transwell insert and cell culture medium (2 mL) was added to the monolayer of cells. After 24 h of incubation, all the wells containing either HA gel alone, HA-DEX, or different concentrations of HA-CSE, but not control cells, were stimulated with LPS at a concentration of 200 ng/mL for further 24 h.

### 4.11. Mutagenicity Assay: Ames Test

The TA100 and TA98 strains of *Salmonella typhimurium* were utilized for the mutagenicity assay in the absence and presence of metabolic activation, i.e., with and without an S9 liver fraction. The tester strains used were selected because they are sensitive and detect a large proportion of known bacterial mutagens and are most commonly used routinely within the pharmaceutical industry [118]. The following specific positive controls were used, respectively, with and without an S9 fraction: 2-Nitrofluorene (2-NF) 2 µg/mL + 4-Nitroquinoline N-oxide (4-NQO) 0.1 µg/mL, and 2-aminoanthracene (2-AA) 5 µg/mL. The final concentration of S9 in the culture was 4.5%.

Approximately 107 bacteria were exposed to 6 concentrations (0.025, 0.050, 0.10, 0.50, 1.0, and 10.0 mg/mL) of CSE, as well as to positive and negative controls, as described before [119].

### 4.12. Statistical Analysis

Experiments were performed in triplicate. Statistical analyses were performed with GraphPad Prism 9.0 software (GraphPad Software, San Diego, CA, USA). Data are presented as mean ± SD and were compared using a one-way ANOVA with an appropriate post hoc test. A *p* value < 0.05 was considered significant.

### 4.13. In Silico Studies

#### Structural Resources and Docking Simulations

The primary structures of the human c-Jun N-terminal kinase 3 (MAPK10) and the NF-κB p65 were retrieved from UniProtKB reviewed (Swiss-Prot) with entry “P53779” and “Q04206”, respectively. The 3D structures of MAPK10 and NF-κB p65 were downloaded from the RCSB Protein Data Bank [120] with PDB code “1PMN” and “1NFI”, respectively.

To optimize the 3D structures of each target for the docking simulation, the potential missing side chains and steric clashes in 3D structures reported in PDB files were added/resolved with molecular modelling using PyMOD3.0 [121,122]. Three-dimensional structures were analyzed and validated with PROCHECK v.3.5.4 [123,124].

The 3D structures of cannflavin A (CID: 10071695), luteolin (CID: 5280445), vitexin (CID: 5280441), genistein (CID: 5280961), cannabidiolic acid (CID: 160570), Δ9-tetrahydrocannabinolic acid (CID: 98523), cannabidiol (CID: 644019), and lucidone B (CID: 14109411) were obtained in sdf format through the PubChem database [125].

The docking simulation was performed using Autodock/VinaXB implemented in the PyMOL2.5 plugin and it was set with an exhaustiveness of 32 and all other parameters as default [126,127]. A box able to enclose the target sensing residues was built with a dimension of 20 Å for each dimension. MGLTOOLS v.1.5.7 scripts [128,129] and OpenBabel v.3.1.0 were used to respectively convert protein and ligand files and to add gasteiger partial charges [130,131]. The interaction network was explored with the P.L.I.P. v. 2.3.0 Tool [132,133].

## 5. Conclusions

The integration of hemp residual biomass into drug production highlights the potential of circular bioeconomy principles to transform industries. By focusing on resource optimization and waste minimization, this approach addresses critical environmental and economic challenges while opening new avenues for sustainable cannabinoid production. As research continues to refine the potential of hemp leaves and other by-products, they could become a cornerstone of the pharmaceutical industry, reducing the environmental footprint of cannabinoid production and fostering a more sustainable future for the *Cannabis* sector. The findings of this study highlight the promising anti-inflammatory properties of CSE extract obtained from *C. sativa* by-products. CSE showed significant downregulation of pro-inflammatory markers in LPS-stimulated RAW 264.7 macrophages without inducing notable cytotoxicity. The extract further demonstrated protective effects in IL-1β stimulated C-20/A4 human immortalized chondrocytes. In silico results confirmed the experimental evidence. However, the primary aim of this study was not to propose our compounds as inhibitors of c-Jun N-terminal kinase 3 (MAPK10) and NF-κB p65, but rather to highlight the potential of CSE to bind within a target sensing region to support in vitro evidence; future experimental investigations will be necessary to further explore such computational evidence.

Incorporating CSE into a hyaluronic acid gel formulation could serve as a blueprint for developing joint inflammation treatments based on sustainable biomass waste, offering both health benefits and promoting a circular bioeconomy. These results underscore the potential of transforming these underutilized *C. sativa* resources into effective therapeutic agents for arthritic diseases.

## Figures and Tables

**Table 1:** TPC, TFC, and antioxidant capacity of CSE.

			Antioxidant Capacity		
	TPC (mg GAE/g)	TFC (mg QE/g)	RP (mg AAE/g)	ABTS (IC50 µg/mL)	DPPH (IC50 µg/mL)
CSE	65.31 ± 2.83	55.18 ± 10.6	58.50 ± 2.4	128.35 ± 3.1	67.93 ± 3.6

Note: Data are expressed as mean ± SD (*n* = 3).

**Table 2:** Most representative matched metabolites in *C. sativa* leaves hydroethanolic extract. Compounds with an area % <0.1 were considered traces.

Name	Retention Time (min)	Formula	Calculated MW	*m*/*z*	Reference Ion	Mass Error (ppm)
Cannabidiolic acid	29.787	C_22_H_30_O_4_	358.21483	357.2076	[M−H]^−^	1.17
Δ^9^-tetrahydrocannabinolic acid	40.725	C_22_H_30_O_4_	358.21456	359.2218	[M+H]^+^	0.41
Cannabidiol	45.903	C_21_H_30_O_2_	314.22572	315.233	[M+H]^+^	3.64
Cannflavin A	40.63	C_26_H_28_O_6_	436.18919	437.1965	[M+H]^+^	1.38
Luteolin	16.598	C_15_H_10_O_6_	286.04839	287.0557	[M+H]^+^	2.27
Vitexin	15.777	C_21_H_20_O_10_	432.10671	433.114	[M+H]^+^	2.45
Genistein	17.997	C_15_H_10_O_5_	270.05291	269.0456	[M−H]^−^	0.33
Lucidone B	40.665	C_24_H_32_O_5_	400.22536	401.2328	[M+H]^+^	0.96

Note: MW, molecular weight.

## Data Availability

The original contributions presented in this study are included in the article/Supplementary Material; further inquiries can be directed to the corresponding author.

## Supplementary Materials

The supporting information can be downloaded at: https://www.mdpi.com/article/10.3390/ijms26020548/s1.

## References

[1] Yan B., Chen Z.S., Hu Y., Yong Q. (2021). Insight in the Recent Application of Polyphenols from Biomass. Front. Bioeng. Biotechnol..

[2] Khayatan D., Nouri K., Momtaz S., Roufogalis B.D., Alidadi M., Jamialahmadi T., Abdolghaffari A.H., Sahebkar A. (2024). Plant-Derived Fermented Products: An Interesting Concept for Human Health. Curr. Dev. Nutr..

[3] Zuo X., Gu Y., Wang C., Zhang J., Zhang J., Wang G., Wang F. (2020). A Systematic Review of the Anti-Inflammatory and Immunomodulatory Properties of 16 Essential Oils of Herbs. Evid. Based Complement. Altern. Med..

[4] Spisni E., Petrocelli G., Imbesi V., Spigarelli R., Azzinnari D., Donati Sarti M., Campieri M., Valerii M.C. (2020). Antioxidant, Anti-Inflammatory, and Microbial-Modulating Activities of Essential Oils: Implications in Colonic Pathophysiology. Int. J. Mol. Sci..

[5] Miguel M.G. (2010). Antioxidant and Anti-Inflammatory Activities of Essential Oils: A Short Review. Molecules.

[6] Chagas M.S.S., Behrens M.D., Moragas-Tellis C.J., Penedo G.X.M., Silva A.R., Gonçalves-de-Albuquerque C.F. (2022). Flavonols and Flavones as Potential Anti-Inflammatory, Antioxidant, and Antibacterial Compounds. Oxid. Med. Cell. Longev..

[7] Palai S., Rudrapal M. (2023). Nanodeliveries of Food Polyphenols as Nutraceuticals. Polyphenols: Food, Nutraceutical, and Nanotherapeutic Applications.

[8] Vamanu E. (2019). Polyphenolic Nutraceuticals to Combat Oxidative Stress Through Microbiota Modulation. Front Pharmacol..

[9] Sahiner M., Yilmaz A.S., Gungor B., Ayoubi Y., Sahiner N. (2022). Therapeutic and Nutraceutical Effects of Polyphenolics from Natural Sources. Molecules.

[10] Zhang Z., Li X., Sang S., McClements D.J., Chen L., Long J., Jiao A., Jin Z., Qiu C. (2022). Polyphenols as Plant-Based Nutraceuticals: Health Effects, Encapsulation, Nano-Delivery, and Application. Foods.

[11] Chávez-Delgado E.L., Jacobo-Velázquez D.A. (2023). Essential Oils: Recent Advances on Their Dual Role as Food Preservatives and Nutraceuticals against the Metabolic Syndrome. Foods.

[12] Matera R., Lucchi E., Valgimigli L. (2023). Plant Essential Oils as Healthy Functional Ingredients of Nutraceuticals and Diet Supplements: A Review. Molecules.

[13] Kaleem M., Ahmad A. (2018). Flavonoids as Nutraceuticals. Therapeutic, Probiotic, and Unconventional Foods.

[14] Bernardini G., Minetti M., Polizzotto G., Biazzo M., Santucci A. (2018). Pro-Apoptotic Activity of French Polynesian Padina Pavonica Extract on Human Osteosarcoma Cells. Mar. Drugs.

[15] Minetti M., Bernardini G., Biazzo M., Gutierrez G., Geminiani M., Petrucci T., Santucci A. (2019). Padina Pavonica Extract Promotes In Vitro Differentiation and Functionality of Human Primary Osteoblasts. Mar. Drugs.

[16] Ubando A.T., Felix C.B., Chen W.-H. (2020). Biorefineries in Circular Bioeconomy: A Comprehensive Review. Bioresour. Technol..

[17] Moscariello C., Matassa S., Esposito G., Papirio S. (2021). From Residue to Resource: The Multifaceted Environmental and Bioeconomy Potential of Industrial Hemp (*Cannabis Sativa* L.). Resour. Conserv. Recycl..

[18] Fordjour E., Manful C.F., Sey A.A., Javed R., Pham T.H., Thomas R., Cheema M. (2023). *Cannabis*: A Multifaceted Plant with Endless Potentials. Front Pharmacol..

[19] Groom Q., Clarke R.C., Merlin M.D. (2014). *Cannabis*: Evolution and Ethnobotany. Plant Ecol. Evol..

[20] Small E. (1975). Morphological Variation of Achenes of *Cannabis*. Can. J. Bot..

[21] Small E., Cronquist A. (1976). A Practical and Natural Taxonomy for *Cannabis*. Taxon.

[22] Clarke R.C., Merlin M.D. (2016). *Cannabis* Domestication, Breeding History, Present-Day Genetic Diversity, and Future Prospects. CRC Crit. Rev. Plant Sci..

[23] Grassi G., McPartland J.M. (2017). Chemical and Morphological Phenotypes in Breeding of *Cannabis Sativa* L. Cannabis sativa L.-Botany and Biotechnology.

[24] Durán-Zuazo V.H., Rodríguez B.C., García-Tejero I.F., Ruiz B.G. (2023). Suitability and Opportunities for *Cannabis Sativa* L. as an Alternative Crop for Mediterranean Environments. Current Applications, Approaches, and Potential Perspectives for Hemp.

[25] Izzo L., Castaldo L., Narváez A., Graziani G., Gaspari A., Rodríguez-Carrasco Y., Ritieni A. (2020). Analysis of Phenolic Compounds in Commercial *Cannabis Sativa* L. Inflorescences Using UHPLC-Q-Orbitrap HRMS. Molecules.

[26] Cicaloni V., Salvini L., Vitalini S., Garzoli S. (2022). Chemical Profiling and Characterization of Different Cultivars of *Cannabis Sativa* L. Inflorescences by SPME-GC-MS and UPLC-MS. Separations.

[27] Lorensen M.D.B.B., Hayat S.Y., Wellner N., Bjarnholt N., Janfelt C. (2023). Leaves of *Cannabis Sativa* and Their Trichomes Studied by DESI and MALDI Mass Spectrometry Imaging for Their Contents of Cannabinoids and Flavonoids. Phytochem. Anal..

[28] Takeda S., Misawa K., Yamamoto I., Watanabe K. (2008). Cannabidiolic Acid as a Selective Cyclooxygenase-2 Inhibitory Component in *Cannabis*. Drug Metab. Dispos..

[29] Kopustinskiene D.M., Masteikova R., Lazauskas R., Bernatoniene J. (2022). *Cannabis Sativa* L. Bioactive Compounds and Their Protective Role in Oxidative Stress and Inflammation. Antioxidants.

[30] Hampson A.J., Grimaldi M., Axelrod J., Wink D. (1998). Cannabidiol and (−)Δ9-Tetrahydrocannabinol Are Neuroprotective Antioxidants. Proc. Natl Acad. Sci. USA.

[31] Burstein S. (2015). Cannabidiol (CBD) and Its Analogs: A Review of Their Effects on Inflammation. Bioorg. Med. Chem..

[32] Wheal A.J., Jadoon K., Randall M.D., O’Sullivan S.E. (2017). In Vivo Cannabidiol Treatment Improves Endothelium-Dependent Vasorelaxation in Mesenteric Arteries of Zucker Diabetic Fatty Rats. Front Pharmacol..

[33] Wheal A.J., Cipriano M., Fowler C.J., Randall M.D., O’Sullivan S.E. (2014). Cannabidiol Improves Vasorelaxation in Zucker Diabetic Fatty Rats through Cyclooxygenase Activation. J. Pharmacol. Exp. Ther..

[34] Rong C., Lee Y., Carmona N.E., Cha D.S., Ragguett R.M., Rosenblat J.D., Mansur R.B., Ho R.C., McIntyre R.S. (2017). Cannabidiol in Medical Marijuana: Research Vistas and Potential Opportunities. Pharmacol. Res..

[35] Atalay S., Jarocka-karpowicz I., Skrzydlewskas E. (2020). Antioxidative and Anti-Inflammatory Properties of Cannabidiol. Antioxidants.

[36] Terkawi M.A., Ebata T., Yokota S., Takahashi D., Endo T., Matsumae G., Shimizu T., Kadoya K., Iwasaki N. (2022). Low-Grade Inflammation in the Pathogenesis of Osteoarthritis: Cellular and Molecular Mechanisms and Strategies for Future Therapeutic Intervention. Biomedicines.

[37] Tsai P.-W., Lee Y.-H., Chen L.-G., Lee C.-J., Wang C.-C. (2018). In Vitro and In Vivo Anti-Osteoarthritis Effects of 2,3,5,4′-Tetrahydroxystilbene-2-O-β-d-Glucoside from Polygonum Multiflorum. Molecules.

[38] O’Brien M., McDougall J.J. (2018). *Cannabis* and Joints: Scientific Evidence for the Alleviation of Osteoarthritis Pain by Cannabinoids. Curr. Opin. Pharmacol..

[39] Deckey D.G., Lara N.J., Gulbrandsen M.T., Hassebrock J.D., Spangehl M.J., Bingham J.S. (2021). Prevalence of Cannabinoid Use in Patients with Hip and Knee Osteoarthritis. J. Am. Acad. Orthop. Surg. Glob. Res. Rev..

[40] Breivik H., Collett B., Ventafridda V., Cohen R., Gallacher D. (2006). Survey of Chronic Pain in Europe: Prevalence, Impact on Daily Life, and Treatment. Eur. J. Pain..

[41] Frane N., Stapleton E., Iturriaga C., Ganz M., Rasquinha V., Duarte R. (2022). Cannabidiol as a Treatment for Arthritis and Joint Pain: An Exploratory Cross-Sectional Study. J. Cannabis Res..

[42] Dunn S.L., Wilkinson J.M., Crawford A., Bunning R.A.D., Le Maitre C.L. (2016). Expression of Cannabinoid Receptors in Human Osteoarthritic Cartilage: Implications for Future Therapies. Cannabis Cannabinoid. Res..

[43] Soliman N., Haroutounian S., Hohmann A.G., Krane E., Liao J., Macleod M., Segelcke D., Sena C., Thomas J., Vollert J. (2021). Systematic Review and Meta-Analysis of Cannabinoids, *Cannabis*-Based Medicines, and Endocannabinoid System Modulators Tested for Antinociceptive Effects in Animal Models of Injury-Related or Pathological Persistent Pain. Pain.

[44] Vela J., Dreyer L., Petersen K.K., Arendt-Nielsen L., Duch K.S., Kristensen S. (2022). Cannabidiol Treatment in Hand Osteoarthritis and Psoriatic Arthritis: A Randomized, Double-Blind, Placebo-Controlled Trial. Pain.

[45] Verrico C.D., Wesson S., Konduri V., Hofferek C.J., Vazquez-Perez J., Blair E., Dunner K., Salimpour P., Decker W.K., Halpert M.M. (2020). A Randomized, Double-Blind, Placebo-Controlled Study of Daily Cannabidiol for the Treatment of Canine Osteoarthritis Pain. Pain.

[46] Rea K.A., Casaretto J.A., Al-Abdul-Wahid M.S., Sukumaran A., Geddes-McAlister J., Rothstein S.J., Akhtar T.A. (2019). Biosynthesis of Cannflavins A and B from *Cannabis Sativa* L. Phytochemistry.

[47] Delgado-Povedano M.M., Sánchez-Carnerero Callado C., Priego-Capote F., Ferreiro-Vera C. (2020). Untargeted Characterization of Extracts from *Cannabis Sativa* L. Cultivars by Gas and Liquid Chromatography Coupled to Mass Spectrometry in High Resolution Mode. Talanta.

[48] Vásquez-Ocmín P.G., Marti G., Bonhomme M., Mathis F., Fournier S., Bertani S., Maciuk A. (2021). Cannabinoids vs. Whole Metabolome: Relevance of Cannabinomics in Analyzing *Cannabis* Varieties. Anal. Chim. Acta..

[49] Sharif O., Bolshakov V.N., Raines S., Newham P., Perkins N.D. (2007). Transcriptional Profiling of the LPS Induced NF-ΚB Response in Macrophages. BMC Immunol..

[50] Scapin G., Patel S.B., Lisnock J., Becker J.W., LoGrasso P.V. (2003). The Structure of JNK3 in Complex with Small Molecule Inhibitors. Chem. Biol..

[51] Shiroma Y., Fujita G., Yamamoto T., Takahashi R., Kumar A., Zhang K.Y.J., Ito A., Osada H., Yoshida M., Tahara H. (2020). Identification of a Selective RelA Inhibitor Based on DSE-FRET Screening Methods. Int. J. Mol. Sci..

[52] Hadley Kershaw E., Hartley S., McLeod C., Polson P. (2021). The Sustainable Path to a Circular Bioeconomy. Trends Biotechnol..

[53] Bioeconomy-Research and Innovation-European Union European Union. https://research-and-innovation.ec.europa.eu/research-area/environment/bioeconomy_en.

[54] Liu Z., de Souza T.S.P., Holland B., Dunshea F., Barrow C., Suleria H.A.R. (2023). Valorization of Food Waste to Produce Value-Added Products Based on Its Bioactive Compounds. Processes.

[55] Squillaci G., Apone F., Sena L.M., Carola A., Tito A., Bimonte M., Lucia A.D., Colucci G., La Cara F., Morana A. (2018). Chestnut (*Castanea sativa* Mill.) Industrial Wastes as a Valued Bioresource for the Production of Active Ingredients. Process Biochem..

[56] Afraz M., Muhammad F., Nisar J., Shah A., Munir S., Ali G., Ahmad A. (2024). Production of Value Added Products from Biomass Waste by Pyrolysis: An Updated Review. Waste Manag. Bull..

[57] Pérez-Marroquín X.A., Estrada-Fernández A.G., García-Ceja A., Aguirre-Álvarez G., León-López A. (2023). Agro-Food Waste as an Ingredient in Functional Beverage Processing: Sources, Functionality, Market and Regulation. Foods.

[58] Jaouhari Y., Travaglia F., Giovannelli L., Picco A., Oz E., Oz F., Bordiga M. (2023). From Industrial Food Waste to Bioactive Ingredients: A Review on the Sustainable Management and Transformation of Plant-Derived Food Waste. Foods.

[59] Osorio L.L.D.R., Flórez-López E., Grande-Tovar C.D. (2021). The Potential of Selected Agri-Food Loss and Waste to Contribute to a Circular Economy: Applications in the Food, Cosmetic and Pharmaceutical Industries. Molecules.

[60] Longo S., Cellura M., Luu L.Q., Nguyen T.Q., Rincione R., Guarino F. (2024). Circular Economy and Life Cycle Thinking Applied to the Biomass Supply Chain: A Review. Renew. Energy.

[61] Fava F., Gardossi L., Brigidi P., Morone P., Carosi D.A.R., Lenzi A. (2021). The Bioeconomy in Italy and the New National Strategy for a More Competitive and Sustainable Country. N. Biotechnol..

[62] Wee Y.B., Berkowitz O., Whelan J., Jost R. (2024). Same, yet Different: Towards Understanding Nutrient Use in Hemp- and Drug-Type *Cannabis*. J. Exp. Bot..

[63] Grassa C.J., Weiblen G.D., Wenger J.P., Dabney C., Poplawski S.G., Timothy Motley S., Michael T.P., Schwartz C.J. (2021). A New *Cannabis* Genome Assembly Associates Elevated Cannabidiol (CBD) with Hemp Introgressed into Marijuana. New Phytol..

[64] Struik P.C., Amaducci S., Bullard M.J., Stutterheim N.C., Venturi G., Cromack H.T.H. (2000). Agronomy of Fibre Hemp (*Cannabis sativa* L.) in Europe. Ind. Crops. Prod..

[65] Blandinières H., Amaducci S. (2022). Adapting the Cultivation of Industrial Hemp (*Cannabis sativa* L.) to Marginal Lands: A Review. GCB Bioenergy.

[66] Haldhar R., Prasad D., Mandal N., Benhiba F., Bahadur I., Dagdag O. (2021). Anticorrosive Properties of a Green and Sustainable Inhibitor from Leaves Extract of *Cannabis sativa* Plant: Experimental and Theoretical Approach. Colloids Surf. Physicochem. Eng. Asp..

[67] Radwan M.M., Chandra S., Gul S., ElSohly M.A. (2021). Cannabinoids, Phenolics, Terpenes and Alkaloids of *Cannabis*. Molecules.

[68] U.S. Food and Drug Administration (2016). Substances Generally Recognized as Safe (Final Rule). Fed. Regist..

[69] Amin I., Mukhrizah O. (2006). Antioxidant Capacity of Methanolic and Water Extracts Prepared from Food-Processing by-Products. J. Sci. Food Agric..

[70] Ahmed M., Ji M., Qin P., Gu Z., Liu Y., Sikandar A., Iqbal M.F., Javeed A. (2019). Phytochemical Screening, Total Phenolic and Flavonoids Contents and Antioxidant Activities of *Citrullus colocynthis* L. and *Cannabis sativa* L. Appl. Ecol. Environ. Res..

[71] Luangpraditkun K., Pimjuk P., Phimnuan P., Wisanwattana W., Wisespongpand C., Waranuch N., Viyoch J. (2024). Anti-Aging Properties of *Cannabis sativa* Leaf Extract against UVA Irradiation. Cosmetics.

[72] Chatzimitakos T., Athanasiadis V., Makrygiannis I., Kalompatsios D., Bozinou E., Lalas S.I. (2024). Bioactive Compound Extraction of Hemp (*Cannabis sativa* L.) Leaves through Response Surface Methodology Optimization. AgriEngineering.

[73] Mkpenie V.N., Essien E.E., Udoh I.I. (2012). Effect of Extraction Conditions on Total Polyphenol Contents, Antioxidant and Antimicrobial Activities of *Cannabis sativa* L. Electron. J. Environ. Agric. Food Chem..

[74] Chen L., Li H.-L., Zhou H.-J., Zhang G.-Z., Zhang Y., Wang Y.-M., Wang M.-Y., Yang H., Gao W. (2024). Feature-Based Molecular Network-Assisted Cannabinoid and Flavonoid Profiling of *Cannabis sativa* Leaves and Their Antioxidant Properties. Antioxidants.

[75] Jin D., Dai K., Xie Z., Chen J. (2020). Secondary Metabolites Profiled in *Cannabis* Inflorescences, Leaves, Stem Barks, and Roots for Medicinal Purposes. Sci. Rep..

[76] Formato M., Crescente G., Scognamiglio M., Fiorentino A., Pecoraro M.T., Piccolella S., Catauro M., Pacifico S. (2020). (–)-Cannabidiolic Acid, a Still Overlooked Bioactive Compound: An Introductory Review and Preliminary Research. Molecules.

[77] Lowin T., Schneider M., Pongratz G. (2019). Joints for Joints: Cannabinoids in the Treatment of Rheumatoid Arthritis. Curr. Opin. Rheumatol..

[78] Wang M., Wang Y.-H., Avula B., Radwan M.M., Wanas A.S., van Antwerp J., Parcher J.F., ElSohly M.A., Khan I.A. (2016). Decarboxylation Study of Acidic Cannabinoids: A Novel Approach Using Ultra-High-Performance Supercritical Fluid Chromatography/Photodiode Array-Mass Spectrometry. Cannabis Cannabinoid Res..

[79] Kim J., Lee C.-G., Hwang S., Yun S.-H., Uprety L.P., Oh K.-I., Singh S., Yoo J., Jeong H., Yong Y. (2022). Anti-Osteoarthritic Effects of Prunella Vulgaris and Gentiana Lutea In Vitro and In Vivo. Antioxidants.

[80] Lu C., Li Y., Hu S., Cai Y., Yang Z., Peng K. (2018). Scoparone Prevents IL-1β-Induced Inflammatory Response in Human Osteoarthritis Chondrocytes through the PI3K/Akt/NF-ΚB Pathway. Biomed. Pharmacother..

[81] Liu C.-C., Zhang Y., Dai B.-L., Ma Y.-J., Zhang Q., Wang Y., Yang H. (2017). Chlorogenic Acid Prevents Inflammatory Responses in IL-1β-Stimulated Human SW-1353 Chondrocytes, a Model for Osteoarthritis. Mol. Med. Rep..

[82] Goldring M.B., Otero M. (2011). Inflammation in Osteoarthritis. Curr. Opin. Rheumatol..

[83] Cadet C., Maheu E. (2021). Non-Steroidal Anti-Inflammatory Drugs in the Pharmacological Management of Osteoarthritis in the Very Old: Prescribe or Proscribe?. Ther. Adv. Musculoskelet. Dis..

[84] Zahan O.-M., Serban O., Gherman C., Fodor D. (2020). The Evaluation of Oxidative Stress in Osteoarthritis. Med. Pharm. Rep..

[85] Duangnin N., Klangjorhor J., Tipparat P., Pinmanee S., Phitak T., Pothacharoen P., Kongtawelert P. (2017). Anti-Inflammatory Effect of Methanol Extracts of Hemp Leaf in IL-1β-Induced Synovitis. Trop. J. Pharm. Res..

[86] Jenei-Lanzl Z., Meurer A., Zaucke F. (2019). Interleukin-1β Signaling in Osteoarthritis–Chondrocytes in Focus. Cell Signal.

[87] Abramson S.B., Attur M., Amin A.R., Clancy R. (2001). Nitric Oxide and Inflammatory Mediators in the Perpetuation of Osteoarthritis. Curr. Rheumatol. Rep..

[88] Ge H., Zou F., Li Y., Liu A., Tu M. (2017). JNK Pathway in Osteoarthritis: Pathological and Therapeutic Aspects. J. Recept. Signal Transduct..

[89] Clancy R., Rediske J., Koehne C., Stoyanovsky D., Amin A., Attur M., Iyama K., Abramson S.B. (2001). Activation of Stress-Activated Protein Kinase in Osteoarthritic Cartilage: Evidence for Nitric Oxide Dependence. Osteoarthr. Cartil..

[90] Choi M.C., Jo J., Park J., Kang H.K., Park Y. (2019). NF-Κb Signaling Pathways in Osteoarthritic Cartilage Destruction. Cells.

[91] Vernarelli J.A., Lambert J.D. (2017). Flavonoid Intake Is Inversely Associated with Obesity and C-Reactive Protein, a Marker for Inflammation, in US Adults. Nutr. Diabetes.

[92] Lefèvre-Arbogast S., Gaudout D., Bensalem J., Letenneur L., Dartigues J.F., Hejblum B.P., Féart C., Delcourt C., Samieri C. (2018). Pattern of Polyphenol Intake and the Long-Term Risk of Dementia in Older Persons. Neurology.

[93] Ciumărnean L., Milaciu M.V., Runcan O., Vesa Ș.C., Răchișan A.L., Negrean V., Perné M.-G., Donca V.I., Alexescu T.-G., Para I. (2020). The Effects of Flavonoids in Cardiovascular Diseases. Molecules.

[94] Lim H., Heo M.Y., Kim H.P. (2019). Flavonoids: Broad Spectrum Agents on Chronic Inflammation. Biomol. Ther..

[95] Werz O., Seegers J., Schaible A.M., Weinigel C., Barz D., Koeberle A., Allegrone G., Pollastro F., Zampieri L., Grassi G. (2014). Cannflavins from Hemp Sprouts, a Novel Cannabinoid-Free Hemp Food Product, Target Microsomal Prostaglandin E2 Synthase-1 and 5-Lipoxygenase. PharmaNutrition.

[96] Barrett M.L., Gordon D., Evans F.J. (1985). Isolation from *Cannabis Sativa* L. of Cannflavin—A Novel Inhibitor of Prostaglandin Production. Biochem. Pharmacol..

[97] Barrett M.L., Scutt A.M., Evans F.J. (1986). Cannflavin A and B, Prenylated Flavones from *Cannabis sativa* L. Experientia.

[98] Koeberle A., Laufer S.A., Werz O. (2016). Design and Development of Microsomal Prostaglandin E _2_ Synthase-1 Inhibitors: Challenges and Future Directions. J. Med. Chem..

[99] Yu Y., Pei F., Li Z. (2022). Orientin and Vitexin Attenuate Lipopolysaccharide-Induced Inflammatory Responses in RAW264.7 Cells: A Molecular Docking Study, Biochemical Characterization, and Mechanism Analysis. Food Sci. Hum. Wellness.

[100] Yang H., Huang J., Mao Y., Wang L., Li R., Ha C. (2019). Vitexin Alleviates Interleukin-1β-induced Inflammatory Responses in Chondrocytes from Osteoarthritis Patients: Involvement of HIF-1α Pathway. Scand. J. Immunol..

[101] Zhang D., Ning T., Wang H. (2022). Vitexin Alleviates Inflammation and Enhances Apoptosis through the Regulation of the JAK/STAT/SOCS Signaling Pathway in the Arthritis Rat Model. J. Biochem. Mol. Toxicol..

[102] Kaux J.-F., Samson A., Crielaard J.-M. (2015). Hyaluronic Acid and Tendon Lesions. Muscles Ligaments Tendons J..

[103] Makvandi P., Della Sala F., di Gennaro M., Solimando N., Pagliuca M., Borzacchiello A. (2022). A Hyaluronic Acid-Based Formulation with Simultaneous Local Drug Delivery and Antioxidant Ability for Active Viscosupplementation. ACS Omega.

[104] Tijani A.O., Thakur D., Mishra D., Frempong D., Chukwunyere U.I., Puri A. (2021). Delivering Therapeutic Cannabinoids via Skin: Current State and Future Perspectives. J. Control. Release.

[105] Pluda S., Beninatto R., Soato M., Barbera C., di Lucia A., Fassina L., Gatti F., Guarise C., Galesso D., Pavan M. (2021). Hyaluronic Acid-Alendronate Conjugate: A Macromolecular Drug Delivery System for Intra-Articular Treatment of Osteoarthritis. Osteoarthr Cart. Open.

[106] d’Angelo I., Provenzano R., Florio E., Lombardi A., Trama U., Ungaro F., Quaglia F., Miro A. (2023). Transmucosal Delivery of the Medical *Cannabis* Oil via a Nanoemulsion Formulation. J. Drug Deliv. Sci. Technol..

[107] Song F.L., Gan R.Y., Zhang Y., Xiao Q., Kuang L., Li H. (2010). Bin Total Phenolic Contents and Antioxidant Capacities of Selected Chinese Medicinal Plants. Int. J. Mol. Sci..

[108] Chang C.-C., Yang M.-H., Wen H.-M., Chern J.-C. (2020). Estimation of Total Flavonoid Content in Propolis by Two Complementary Colometric Methods. J. Food Drug Anal..

[109] Jayaprakasha G.K., Singh R.P., Sakariah K.K. (2001). Antioxidant Activity of Grape Seed (*Vitis vinifera*) Extracts on Peroxidation Models in Vitro. Food Chem..

[110] Ilyasov I.R., Beloborodov V.L., Selivanova I.A., Terekhov R.P. (2020). ABTS/PP Decolorization Assay of Antioxidant Capacity Reaction Pathways. Int. J. Mol. Sci..

[111] Yen G.-C., Chen H.-Y. (1995). Antioxidant Activity of Various Tea Extracts in Relation to Their Antimutagenicity. J. Agric. Food Chem..

[112] Frusciante L., Geminiani M., Shabab B., Olmastroni T., Scavello G., Rossi M., Mastroeni P., Nyong’a C.N., Salvini L., Lamponi S. (2024). Exploring the Antioxidant and Anti-Inflammatory Potential of Saffron (*Crocus sativus*) Tepals Extract within the Circular Bioeconomy. Antioxidants.

[113] Frusciante L., Geminiani M., Trezza A., Olmastroni T., Mastroeni P., Salvini L., Lamponi S., Bernini A., Grasso D., Dreassi E. (2024). Phytochemical Composition, Anti-Inflammatory Property, and Anti-Atopic Effect of *Chaetomorpha linum* Extract. Mar Drugs.

[114] Ng N., Ooi L. (2021). A Simple Microplate Assay for Reactive Oxygen Species Generation and Rapid Cellular Protein Normalization. Bio. Protoc..

[115] Feoktistova M., Geserick P., Leverkus M. (2016). Crystal Violet Assay for Determining Viability of Cultured Cells. Cold Spring Harb. Protoc..

[116] Mastroeni P., Geminiani M., Olmastroni T., Frusciante L., Trezza A., Visibelli A., Santucci A. (2024). An in Vitro Cell Model for Exploring Inflammatory and Amyloidogenic Events in Alkaptonuria. J. Cell. Physiol..

[117] Mastroeni P., Trezza A., Geminiani M., Frusciante L., Visibelli A., Santucci A. (2024). HGA Triggers SAA Aggregation and Accelerates Fibril Formation in the C20/A4 Alkaptonuria Cell Model. Cells.

[118] Mortelmans K., Zeiger E. (2000). The Ames Salmonella/Microsome Mutagenicity Assay. Mutat. Res. Fundam. Mol. Mech. Mutagen..

[119] Frusciante L., Geminiani M., Olmastroni T., Mastroeni P., Trezza A., Salvini L., Lamponi S., Spiga O., Santucci A. (2024). Repurposing *Castanea sativa* Spiny Burr By-Products Extract as a Potentially Effective Anti-Inflammatory Agent for Novel Future Biotechnological Applications. Life.

[120] Berman H.M., Westbrook J., Feng Z., Gilliland G., Bhat T.N., Weissig H., Shindyalov I.N., Bourne P.E. (2000). The Protein Data Bank. Nucleic. Acids Res..

[121] Janson G., Paiardini A. (2021). PyMod 3: A Complete Suite for Structural Bioinformatics in PyMOL. Bioinformatics.

[122] Trezza A., Barletta R., Geminiani M., Frusciante L., Olmastroni T., Sannio F., Docquier J.-D., Santucci A. (2024). Chestnut Burrs as Natural Source of Antimicrobial Bioactive Compounds: A Valorization of Agri-Food Waste. Appl. Sci..

[123] Laskowski R.A., MacArthur M.W., Moss D.S., Thornton J.M. (1993). PROCHECK: A Program to Check the Stereochemical Quality of Protein Structures. J. Appl. Crystallogr..

[124] Trezza A., Geminiani M., Cutrera G., Dreassi E., Frusciante L., Lamponi S., Spiga O., Santucci A. (2024). A Drug Discovery Approach to a Reveal Novel Antioxidant Natural Source: The Case of Chestnut Burr Biomass. Int. J. Mol. Sci..

[125] Kim S., Chen J., Cheng T., Gindulyte A., He J., He S., Li Q., Shoemaker B.A., Thiessen P.A., Yu B. (2023). PubChem 2023 Update. Nucleic Acids Res..

[126] Koebel M.R., Schmadeke G., Posner R.G., Sirimulla S. (2016). AutoDock VinaXB: Implementation of XBSF, New Empirical Halogen Bond Scoring Function, into AutoDock Vina. J. Cheminform..

[127] Pessina F., Gamberucci A., Chen J., Liu B., Vangheluwe P., Gorelli B., Lorenzini S., Spiga O., Trezza A., Sgaragli G. (2018). Negative Chronotropism, Positive Inotropism and Lusitropism of 3,5-Di-t-Butyl-4-Hydroxyanisole (DTBHA) on Rat Heart Preparations Occur through Reduction of RyR2 Ca^2+^ Leak. Biochem. Pharmacol..

[128] Fusi F., Durante M., Spiga O., Trezza A., Frosini M., Floriddia E., Teodori E., Dei S., Saponara S. (2016). In Vitro and in Silico Analysis of the Vascular Effects of Asymmetrical N, N-Bis(Alkanol)Amine Aryl Esters, Novel Multidrug Resistance-Reverting Agents. Naunyn Schmiedebergs Arch. Pharmacol..

[129] Morris G.M., Huey R., Lindstrom W., Sanner M.F., Belew R.K., Goodsell D.S., Olson A.J. (2009). AutoDock4 and AutoDockTools4: Automated Docking with Selective Receptor Flexibility. J. Comput. Chem..

[130] O’Boyle N.M., Banck M., James C.A., Morley C., Vandermeersch T., Hutchison G.R. (2011). Open Babel: An Open Chemical Toolbox. J. Cheminform..

[131] Cuong N.M., Son N.T., Nhan N.T., Khanh P.N., Huong T.T., Tram N.T.T., Sgaragli G., Ahmed A., Trezza A., Spiga O. (2020). Vasorelaxing Activity of R-(−)-3′-Hydroxy-2,4,5-Trimethoxydalbergiquinol from Dalbergia Tonkinensis: Involvement of Smooth Muscle CaV1.2 Channels. Planta Med..

[132] Adasme M.F., Linnemann K.L., Bolz S.N., Kaiser F., Salentin S., Haupt V.J., Schroeder M. (2021). PLIP 2021: Expanding the Scope of the Protein–Ligand Interaction Profiler to DNA and RNA. Nucleic Acids Res..

[133] Trezza A., Spiga O., Mugnai P., Saponara S., Sgaragli G., Fusi F. (2022). Functional, Electrophysiology, and Molecular Dynamics Analysis of Quercetin-Induced Contraction of Rat Vascular Musculature. Eur. J. Pharmacol..

