# Supplementary material for: Enhancing Industrial Hemp (Cannabis sativa) Leaf By-Products: Bioactive Compounds, Anti-Inflammatory Properties, and Potential Health Applications
Source: Int J Mol Sci. 2025 Jan 10;26(2):548. doi: 10.3390/ijms26020548 (PMC11765263; doi:10.3390/ijms26020548)
Supplement: Supplementary file 1 [file ijms-26-00548-s001.zip › ijms-3365776-supplementary.pdf]

Table S1: Complete list of metabolites found in CSE

Name	Retention time (min)	Formula	Calculated MW	*m/z*	Reference ion	Mass error (ppm)	Peak Area (%)
cannabidiolic acid	29.787	C22H30O4	358.21483	357.2076	[M-H]-	1.17	78.92
delta9-tetrahydrocannabinolic acid	40.725	C22H30O4	358.21456	359.2218	[M+H]+	0.41	11.91
CBD	45.903	C21H30O2	314.22572	315.233	[M+H]+	3.64	3.56
Cannflavin A	40.63	C26H28O6	436.18919	437.1965	[M+H]+	1.38	1.45
Luteolin	16.598	C15H10O6	286.04839	287.0557	[M+H]+	2.27	1.09
Vitexin	15.777	C21H20O10	432.10671	433.114	[M+H]+	2.45	0.63
Genistein	17.997	C15H10O5	270.05291	269.0456	[M-H]-	0.33	0.57
Lucidone B	40.665	C24H32O5	400.22536	401.2328	[M+H]+	0.96	0.20
CBNA	34.141	C22H26O4	354.1833	355.19055	[M+H]+1	0.55	Tr.
TETRAHYDROCANNABIVARIN	37.627	C19H26O2	286.19325	285.18598	[M-H]-1	-0.1	Tr.
Kaempferol	21.024	C15H10O6	286.04842	285.04104	[M-H]-1	2.4	Tr.
Cannabidivarin	38.157	C19H26O2	286.19411	287.20139	[M+H]+1	2.9	Tr.
DELTA-9-CIS-TETRAHYDROCANNABINOL. (-)-	40.587	C21H30O2	314.22468	313.21741	[M-H]-1	0.33	Tr.
4,5-Dihydroxy-3,7-dimethoxy-9,10-dihydrophenanthrene	30.337	C16H16O4	272.10517	271.09775	[M-H]-1	1.16	Tr.
Quercetin 3-O-.alpha.-L-arabinopyranside	18.189	C20H18O11	434.08464	435.09192	[M+H]+1	-0.62	Tr.
Chlorogenic acid	9.578	C16H18O9	354.09419	355.10147	[M+H]+1	-2.51	Tr.
8,8'-lignan-3,3',4,4',5-tetra-me ether	38.654	C22H30O5	374.21004	375.21732	[M+H]+1	1.91	Tr.
Linalyl oxide	47.211	C10H18O2	170.13118	171.13846	[M+H]+1	2.95	Tr.
Cannabispirone	40.72	C15H18O3	246.12605	247.13333	[M+H]+1	1.87	Tr.
Caffeoyl tyramine	16.242	C17H17NO4	299.11595	298.10868	[M-H]-1	0.65	Tr.
Flavosativaside	2.713	C27H30O15	594.15956	595.16656	[M+H]+1	1.83	Tr.
Cannabinol	36.477	C21H26O2	310.19368	309.18641	[M-H]-1	1.3	Tr.
5-dodecylbenzene-1.3-diol isomer (phenol derivative)	35.317	C18H28O2	276.20952	275.20179	[M-H]-1	2.13	Tr.
(7aR,13aR)-6,7a-Dihydroxy-9,10-dimethoxy-3,3-dimethyl-13,13a-dihydro-3H-chromeno[3,4-b]pyrano[2,3-h]chromen-7(7aH)-one	2.656	C23H22O8	426.13075	427.13803	[M+H]+1	-1.67	Tr.
3.4-dihydro-7-(4-hydroxyphenyl)-2,2-dimethyl-2H-1-benzopyran-3,5-diol	21.956	C17H18O4	286.12129	287.12857	[M+H]+1	2.74	Tr.
Cannithrene 1/Cannabispiradienone/Isocannabispiradienone	24.721	C15H14O3	242.09418	241.08691	[M-H]-1	-0.46	Tr.
Apigenin	15.389	C15H10O5	270.05333	271.06061	[M+H]+1	1.88	Tr.
Chrysoeriol	23.595	C16H12O6	300.06367	299.05639	[M-H]-1	0.92	Tr.
CBL	35.159	C21H26O2	310.1938	309.18652	[M-H]-1	1.67	Tr.
1,2-Benzenediol, 5-(3-(2,4-dihydroxyphenyl)propyl)-3,4-bis(3-methyl-2-butenyl)-	44.997	C25H32O4	396.22917	397.23645	[M+H]+1	-2.24	Tr.

CBGA	42.633	C22H32O4	360.23083	359.2235	[M-H]-1	2.15	Tr.
Glycitin	14.477	C22H22O10	446.1223	445.11502	[M-H]-1	2.24	Tr.
(-)-7-Hydroxycannabichromane	34.645	C21H32O3	332.23542	331.22814	[M-H]-1	0.82	Tr.
Cannabispirenol A/Cannabispirenone A e B/3,4'-Dihydroxy-5-methoxybibenzyl	37.63	C15H16O3	244.10942	243.10214	[M-H]-1	-2.15	Tr.
alpha-Cannabispiranol/beta-Cannabispiranol	27.841	C15H20O3	248.14095	249.14822	[M+H]+1	-1.2	Tr.
CBON isomer	36.023	C21H28O3	328.20482	329.2121	[M+H]+1	2.97	Tr.
(-)-7R-Cannabicoumarononic acid A	38.569	C22H28O5	372.19469	373.20197	[M+H]+1	2.73	Tr.
Juniperic acid	45.554	C16H32O3	272.23556	271.22828	[M-H]-1	1.53	Tr.
4-(6-Hydroxy-1,2,3,9a-tetrahydro-3aH-spiro[cyclopenta[b]chromene-9,1'-cyclopentan]-3a-yl)-1,3-benzenediol	30.636	C22H24O4	352.16838	353.17566	[M+H]+1	2.62	Tr.
Isocannabispiran	26.348	C15H18O3	246.12595	245.11849	[M-H]-1	1.44	Tr.
Tetrahydrocannabivarinic acid	32.16	C20H26O4	330.18401	331.19128	[M+H]+1	2.72	Tr.
Cannabivarin	33.086	C19H22O2	282.16207	281.15479	[M-H]-1	0.32	Tr.
Dihydroresveratrol	27.725	C14H14O3	230.09409	229.08682	[M-H]-1	-0.87	Tr.
Taxifolin	15.197	C15H12O7	304.05916	303.05188	[M-H]-1	2.82	Tr.
isoquercetin	16.95	C21H20O12	464.09485	463.08757	[M-H]-1	-1.35	Tr.
CBL-Me ester	37.47	C23H32O4	372.22999	371.22272	[M-H]-1	-0.18	Tr.
CBG	41.554	C21H32O2	316.24068	317.24796	[M+H]+1	1.42	Tr.
(±)-Naringenin	28.752	C15H12O5	272.06885	271.06157	[M-H]-1	1.38	Tr.
3,5,7-trihydroxy-2-phenyl-4H-chromen-4-one	16.009	C15H10O5	270.05358	271.06085	[M+H]+1	2.79	Tr.
Embelin	34.401	C17H26O4	294.18376	293.17648	[M-H]-1	2.21	Tr.
11-hydroxytephrosin	13.31	C23H22O8	426.13039	427.13766	[M+H]+1	-2.53	Tr.
Eugenol	19.193	C10H12O2	164.08365	165.09093	[M+H]+1	-0.48	Tr.
(+-)-Demethylgrossamide/Cannabisin C	19.721	C35H34N2O8	610.23066	609.22338	[M-H]-1	-1.41	Tr.
D8-THC	35.357	C21H30O2	314.22514	313.21786	[M-H]-1	1.77	Tr.
5-deoxystrigol	26.666	C19H22O5	330.1465	331.15378	[M+H]+1	-0.67	Tr.
(E)-Ferulic acid	15.67	C10H10O4	194.0584	195.06567	[M+H]+1	2.51	Tr.
Cannabigerol	43.283	C21H32O2	316.24085	315.23358	[M-H]-1	1.97	Tr.
11-Nor-9-carboxy-thc	31.964	C21H28O4	344.19935	343.19208	[M-H]-1	1.72	Tr.
Rutin	15.74	C27H30O16	610.15401	609.14673	[M-H]-1	1.02	Tr.
Cannabisol derivative	30.025	C22H30O2	326.22532	325.21805	[M-H]-1	2.28	Tr.
Divarinic acid	10.774	C10H12O4	196.07334	197.08061	[M+H]+1	-1.14	Tr.
Cannabidivarinic acid	33.981	C20H26O4	330.1834	331.19067	[M+H]+1	0.87	Tr.
Eriodictyol	19.408	C15H12O6	288.0641	287.05682	[M-H]-1	2.47	Tr.
Diosmetin	15.104	C16H12O6	300.06295	299.05567	[M-H]-1	-1.46	Tr.
CBC	49.072	C21H30O2	314.22474	313.21747	[M-H]-1	0.52	Tr.
Orientin	18.631	C21H20O11	448.10036	447.09308	[M-H]-1	-0.45	Tr.

5,7,4'-Trihydroxy-3,6-dimethoxy-3',5'-diprenylflavone	34.673	C27H30O7	466.19898	465.1917	[M-H]-1	-0.38	Tr.
Cannabisin B	16.221	C34H32N2O8	596.21492	595.20764	[M-H]-1	-1.59	Tr.
QUERCETIN 3,7-DIGLUCOSIDE	14.586	C27H30O17	626.14949	625.14221	[M-H]-1	1.9	Tr.
Quercitrin	14.02	C21H20O11	448.10124	447.09396	[M-H]-1	1.52	Tr.
Cannflavin B	29.267	C21H20O6	368.12547	369.13275	[M+H]+1	-1.39	Tr.
CBLA-derivative	30.046	C22H28O3	340.20442	341.2117	[M+H]+1	1.7	Tr.
Kaempferol-3-Glucuronide	12.117	C21H18O12	462.07908	461.07181	[M-H]-1	-1.6	Tr.
Cytisoside	22.097	C22H22O10	446.1221	445.11482	[M-H]-1	1.79	Tr.
cannabistilbene 1	27.554	C20H24O3	312.17317	311.16589	[M-H]-1	2	Tr.
2,3,5,6-tetramethoxy-9,10-dihydrophenanthrene-1,4-dione	26.509	C18H18O6	330.1098	329.10252	[M-H]-1	-1.64	Tr.
CBG derivative	43.284	C22H30O3	342.21998	341.21271	[M-H]-1	1.43	Tr.
Carexane P-isomer 1/Carexane P-isomer 2	32.73	C20H22O3	310.15768	309.1504	[M-H]-1	2.52	Tr.
Hildgardtol B	11.577	C21H22O4	338.15224	339.15952	[M+H]+1	1.27	Tr.
Wighteone	33.448	C20H18O5	338.11635	337.10907	[M-H]-1	2.73	Tr.
6,8-Diprenylnaringenin	21.145	C25H28O5	408.19346	407.18619	[M-H]-1	-0.51	Tr.
(2E)-3-(4-hydroxyphenyl)-N-(2-phenylethyl)prop-2-enimidic acid	24.953	C17H17NO2	267.12599	266.11871	[M-H]-1	0.23	Tr.
2-Hydroxy-3-methyl-9H-carbazole	8.316	C13H11NO	197.08453	198.0918	[M+H]+1	2.35	Tr.
7-Hydroxy-3-(4-hydroxyphenyl)-4-oxo-4H-chromen-8-yl hexopyranoside	18.269	C21H20O10	432.10618	431.09891	[M-H]-1	1.24	Tr.
Grossamide	22.262	C36H36N2O8	624.24782	623.24054	[M-H]-1	1.04	Tr.
Acuminatin	30.606	C21H24O4	340.16777	339.16049	[M-H]-1	0.91	Tr.
Rhoifolin	22.658	C27H30O14	578.1652	577.15792	[M-H]-1	2.84	Tr.
Sucrose	17.526	C12H22O11	342.11714	341.10986	[M-H]-1	2.71	Tr.

Tr.= Traces (Peak area <0.1%)
